# EGFR‐STAT3 activation provides a therapeutic rationale for targeting aggressive ETV1‐positive prostate cancer

**DOI:** 10.1002/1878-0261.70069

**Published:** 2025-08-14

**Authors:** Elsa Gomes Paiva, Bernardo Orr, Ana Azeredo, Andreia Brandão, Manuel R. Teixeira, Paula Paulo

**Affiliations:** ^1^ Cancer Genetics Group, IPO Porto Research Center (CI‐IPOP)/RISE@CI‐IPOP (Health Research Network) Portuguese Oncology Institute of Porto (IPO Porto)/Porto Comprehensive Cancer Center Portugal; ^2^ PhD Program in Biomedical Sciences, School of Medicine and Biomedical Sciences (ICBAS) University of Porto Portugal; ^3^ Master Program in Oncology, School of Medicine and Biomedical Sciences (ICBAS) University of Porto Portugal; ^4^ Department of Laboratory Genetics Portuguese Oncology Institute of Porto (IPO Porto)/Porto Comprehensive Cancer Center Raquel Seruca (Porto.CCC Raquel Seruca) Portugal; ^5^ School of Medicine and Biomedical Sciences (ICBAS) University of Porto Portugal

**Keywords:** EGFR/STAT3 activation, Erlotinib, ETV1 rearrangements/overexpression, feedback loop, prostate cancer, TTI‐101

## Abstract

Prostate cancer (PCa) is the fifth leading cause of cancer‐related death. The lack of data linking genomic alterations to targeted treatment strategies has hindered progress in disease management. Genomic rearrangements involving the ETS transcription factors *ERG* or *ETV1* are among the most frequent genetic alterations in PCa; however, their clinical utility remains elusive. Using PCa cells overexpressing ETV1 or ERG, representing early and advanced disease stages, we unveiled a positive feedback loop between ETV1 and EGFR, with STAT3 acting as a downstream effector of ETV1–EGFR signaling. Analysis of external datasets revealed that both EGFR and STAT3 are significantly upregulated in ETV1‐positive PCa, consistent with ChIP‐seq data identifying them as direct ETV1 targets. Accordingly, combined inhibition of EGFR and STAT3 using Erlotinib and TTI‐101, respectively, led to a significant reduction in 2D and 3D cell growth of early and advanced PCa cells overexpressing ETV1. Collectively, our findings highlight EGFR–STAT3 activation as a novel ETV1‐regulated oncogenic pathway, providing a rationale for repurposing EGFR inhibitors in combination with STAT3 inhibitors as a therapeutic strategy for the 8–10% of prostate carcinomas characterized by ETV1 rearrangements/overexpression.

## Graphical abstract

Cotargeting EGFR and STAT3 with Erlotinib and TTI‐101 impairs both 2D and 3D growth of ETV1‐overexpressing prostate cancer cells by disrupting a self‐sustaining ETV1–EGFR positive feedback loop that promotes EGFR and STAT3 expression and phosphorylation (activation). In contrast, ERG‐overexpressing cells show reduced EGFR–STAT3 activation and consequently a lower therapeutic response to Erlotinib and TTI‐101.

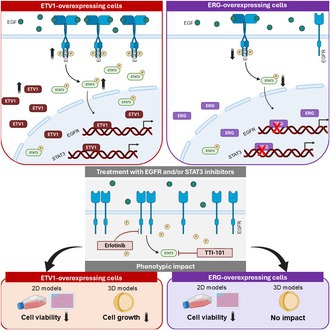

**Abbreviations**
EGFR: epidermal growth factor receptor
ERG: ETS related gene
ETV1: ETS Variant 1
FDA: Food and Drug Administration
HER: human epidermal receptor
HSA: highest single agent
HTS: high‐throughput screening
JAK/STAT: Janus kinase/signal transducers and activators of transcription
PCa: prostate cancer
PEA3: polyoma enhancer activator 3
PI: propidium iodide
STATs: signal transducers and activators of transcription

## 1 Introduction

Prostate cancer (PCa) has the second highest incidence rate in men and is the fifth leading cause of male cancer mortality worldwide [1, 2]. While risk factors such as age, ethnicity, and family history are well‐established, the genetic landscape of PCa remains complex and scarcely understood. Despite the availability of antiandrogen therapies, which allow extending the survival of nonorgan confined PCa, there is currently a lack of targeted treatment options for metastatic androgen‐resistant disease. The presence of genetic rearrangements involving different members of the ETS family of transcription factors underscores distinct PCa molecular subtypes [3] and highlights the need for deeper exploration into how specific genetic alterations can inform more effective treatment strategies for PCa patients. The ETS related gene (ERG) subfamily, composed of *ERG*, *FLI1*, and *FEV* genes, and the Polyoma Enhancer Activator 3 (PEA3) subfamily, composed of the *ETV1*, *ETV4*, and *ETV5* genes, are the best studied ETS subfamilies in PCa [4, 5]. *ERG* rearrangements are found in 40–50% of the prostate carcinomas, *ETV1* rearrangements/overexpression in 8–10%, and rearrangements involving *ETV4*, *ETV5*, or *FLI1* in 2–5% of the cases, being mutually exclusive [4].

ETS rearrangements are considered early molecular events in prostate carcinogenesis, but they are equally prevalent in advanced PCa stages, including metastatic cancer [6, 7]. Although ERG rearrangements are frequently found in PCa, their clinical relevance remains controversial due to inconsistent prognostic value across studies [8]. In contrast, ETV1 overexpression, though less frequent, has been consistently linked to higher disease aggressiveness, poorer outcomes, and increased cellular invasion [9, 10, 11]. We, and others, have identified both specific and shared downstream targets of ERG and ETV1 in prostate carcinomas, supporting the existence of targetable molecular pathways that may overcome the challenges in direct targeting ETS transcription factors as a therapeutic approach [12, 13].

The Epidermal Growth Factor Receptor (EGFR), also known as HER1 or ERBB1, belongs to the human epidermal receptor (HER) family, along with HER2, HER3, and HER4 [14]. Several extracellular ligands can bind and activate EGFR, triggering intracellular phosphorylation reactions that induce multiple downstream signaling cascades, crucial for cell proliferation, survival, and migration [14, 15]. In many cancers, including PCa, oncogenic mutations, amplification, and overexpression of EGFR are common, contributing to tumor development, progression, and metastasis [14]. Recently, several links between EGFR function and PCa progression have been proposed, including genomic instability, metabolic reprogramming, and dietary influences, which underscore the importance of targeting EGFR and its associated pathways in developing effective treatments for PCa [16, 17, 18]. One of the downstream targets of EGFR signaling is the activation of the Janus kinase/signal transducers and activators of transcription (JAK/STAT) pathway. Increased JAK activation enhances activation of Signal Transducers and Activators of Transcription (STATs), which in turn alter the expression of genes involved in cellular proliferation, angiogenesis, and apoptosis. Abnormal activation of STAT1, STAT3, STAT5A, and STAT5B has been found in multiple carcinomas and cancer cell lines and linked to inflammation, invasion, metastasis, and poor prognosis [19, 20, 21, 22, 23]. In PCa, STAT3 and STAT5 are frequently found overexpressed in lymph nodes and bone metastasis, and associated with higher pathological staging and antiandrogen resistance [24]. More recently, STAT3 has been associated with metastatic behavior [25] and identified as a target for sensitizing PCa cells to irradiation [26].

In light of the availability of EGFR inhibitors currently approved by the Food and Drug Administration (FDA) for the treatment of several carcinomas, namely, head and neck squamous cell carcinomas (HNSCC), nonsmall cell lung cancer (NSCLC) with specific EGFR mutations, advanced pancreatic cancer, and metastatic colorectal carcinomas without activating *KRAS*/*BRAF* mutations [27], we questioned whether EGFR‐mediated STAT3 activation could underlie PCa aggressiveness in a specific ETS molecular subtype, opening a therapeutic window for EGFR and/or STAT3 inhibitors. Despite not yet being used in clinical practice, several STAT3 inhibitors have demonstrated a reduction of malignant phenotypes in different cancer types [28, 29, 30, 31], with AZD9150 and TTI‐101 (formerly C188‐9) showing promising therapeutic efficacy in clinical trials [32, 33, 34, 35].

In this study, we explored whether EGFR/STATs activation could underlie, at least in part, the oncogenic behavior of PCa cells harboring ETV1 overexpression, as well as the antioncogenic potential of EGFR/STATs inhibition in ETS‐positive PCa cells. Using cell models mimicking early and advanced PCa, we demonstrate that ERG and ETV1 differentially regulate the activation of EGFR, STAT3, and STAT5A in prostate cells, which modulates sensitivity to Erlotinib (EGFR inhibitor) and TTI‐101 (STAT3 inhibitor) synergistic inhibition in both 2D and 3D PCa cell models. Collectively, our results identify a novel, ETV1‐dependent, oncogenic pathway in prostate cells and highlight the potential therapeutic outcome of coinhibition of EGFR and STAT3 in prostate carcinomas with ETV1 overexpression.

## 2 Materials and methods

### 2.1 Cell culture

We used PNT2‐derived cells with *de novo* ERG and ETV1 overexpression as early‐stage models of PCa, and LNCaP and VCaP cells as advanced PCa cell lines with underlying *ETV1* and *ERG* rearrangements/overexpression, respectively. PNT2 (RRID: CVCL_2164) and VCaP (RRID: CVCL_2235) cells were formerly obtained from the European Collection of Cell Cultures (Sigma‐Aldrich, St. Louis, MO, USA), while LNCaP cells (RRID: CVCL_0395) were obtained from the German Resource Centre for Biological Material (DSMZ, Braunschweig, Germany).

Cell line authenticity was validated by G‐band karyotyping at the Department of Laboratory Genetics of the Portuguese Oncology Institute of Porto at the beginning of the study, and cell derivation was controlled by using low‐passage cell stocks.

PNT2‐derived clonal cell populations with *de novo* overexpression of *ETV1* or *ERG* and LNCaP‐derived cell populations with stable ETV1 silencing were previously established [12]. All cell lines were grown in a humidified chamber at 37 °C with 5% CO_2_. PNT2‐ and LNCaP‐derived cell populations were grown in RPMI‐1640 medium (ThermoFisher Scientific™, Waltham, MA, USA) and VCaP cells were grown in DMEM medium (PAN‐Biotech GmbH, Aidenbach, Germany). All media were supplemented with 10% Fetal Bovine Serum (FBS) and 1% Penicillin/Streptomycin (ThermoFisher Scientific™). To induce EGFR activation, cells were serum‐starved for 24 h, and 1 μg of EGF (ThermoFisher Scientific™) was added to T75 culture flasks containing 5–10 × 10^6^ cells for 20 min before proceeding to total protein extraction.

Cell lines were regularly tested for contaminations with *Mycoplasma spp*., using the TaKaRa PCR Mycoplasma Detection Set (Takara Bio, Inc., Kusatsu, Shiga, Japan).

### 2.2 Western blotting

To obtain total protein extracts, 200 μL of RIPA lysis buffer (Santa Cruz Biotechnology, Dallas, TX, USA) was used to scrape cells from T75 flasks. Lysates were collected and passed through a 20‐gauge needle. The cell lysate was set on ice for 10 min, followed by centrifugation at 18 000 ***g*** for 10 min at 4 °C to obtain a clean protein extract (supernatant). For protein quantification, the Qubit™ 4 Fluorometer and the Qubit™ Protein Broad Range Assay Kit, both from ThermoFisher Scientific™, were used following the manufacturer's recommendations. For protein separation, 10% SDS/polyacrylamide gel electrophoresis (SDS/PAGE) was used. For each sample, 30 to 60 μg of total proteins were analyzed. After electrophoresis, the proteins were transferred to a 0.22 μm nitrocellulose membrane, using the Trans‐Blot Turbo Transfer System (Bio‐Rad Laboratories, Hercules, CA, USA), following the manufacturer's recommendations. The primary antibodies used were anti‐mouse EGFR (#66455‐1‐Ig; Proteintech, Manchester, UK; 1 : 10 000), anti‐mouse STAT3 (Abcam, Cambridge, MA, USA; 1 : 2000), anti‐rabbit STAT5A (#ab32043; Abcam; 1 : 1000), anti‐rabbit phospho‐EGFR Tyr1068 (#ab40815; Abcam; 1 : 3000), anti‐rabbit phospho‐STAT3 Tyr705 (#9131S; Cell Signaling, Danvers, MA, USA; 1 : 500), anti‐rabbit phospho‐STAT5A Tyr694 (#ab32364; Abcam; 1 : 3000), anti‐mouse ETV1 (#SAB1403794; Sigma‐Aldrich; 1 : 2000), and anti‐mouse β‐actin (#10684‐1‐AP; Sigma‐Aldrich; 1 : 8000). The secondary antibodies used were goat anti‐rabbit (#sc‐2359; Santa Cruz Biotechnology, 1 : 10 000) and goat anti‐mouse (#1721011; Bio‐Rad Laboratories, 1 : 2500). Proteins were detected by chemiluminescence using the Clarity™ Western ECL Substrate (Bio‐Rad Laboratories), and bands were developed by autoradiography. The relative quantification of proteins obtained from western blots was performed using imagej (v.2), available from the National Institutes of Health (Bethesda, MD, USA). For each protein, the defined size of the ROI was maintained between the different lanes for each cell line. The pixel density in the bands was measured using the Integrated Density (IntDen) option. Background values were subtracted from values obtained from protein bands, and the densitometry analysis for each protein was normalized to control samples. GraphPad Prism software was used for statistical analysis and data plotting. All western blots were performed in triplicate unless otherwise specified.

### 2.3 *In silico* analysis of ETV1‐regulated genes in publicly available data from chromatin immunoprecipitation (ChIP) experiments

To verify whether ETV1 directly regulates the expression of EGFR, STAT3, and STAT5A, we used publicly available Chromatin Immunoprecipitation data from GEO DataSets, from the National Center for Biotechnology Information (NCBI). Two studies used nonconventional approaches to identify ETV1‐bound chromatin regions: GSE39388 in LNCaP [9] and GSE29808 in RWPE1‐ETV1 [36] cell models. In both studies, ERG‐bound chromatin regions were also studied. In the first study, target genes were identified using the Affymetrix Human Promoter 1.0R Array. The genomic region defined as the promoter region was at a distance of 8000–2000 bp from the transcription start site, and both genomic positions and nearby coding genes were reported according to NCBI Human Genome Assembly Build 36 (hg18) (GSE39388) [9]. EGFR, STAT3, and STAT5A were browsed in published target gene lists classified into three subsets, namely, ERG‐ETV1 common targets, ERG‐targets only, and ETV1‐targets only. In the second study, target genes were identified through next‐generation sequencing, and genomic coordinates were outputted using the NCBI Human Genome Assembly Build 36 (hg18) (GSE29808) [36]. To determine associations with our genes of interest, genomic coordinates were imported into the UCSC Genome Browser website (https://genome.ucsc.edu/) and the browser was used to identify a match between the ChIP‐seq regions and the promoter regions of EGFR, STAT3, and STAT5A.

### 2.4 *In silico* analysis of EGFR and STAT3 expression in publicly available microarray expression data

To validate the association between EGFR/STAT3 and ERG/ETV1 expression in our cell models, we used publicly available expression arrays (GEO DataSets, NCBI) from RWPE‐1 cells with *de novo* overexpression of ETV1 or ERG (GSE29438 and GSE39388), and from LNCaP and VCaP cells with ETV1 or ERG silencing, respectively (GSE39388). ProbeSets for EGFR and STAT3 in each microarray platform were identified using the Gemma Database (https://gemma.msl.ubc.ca [37]) and sample values were obtained using GEO2R. In GSE29438 [based on Nimblegen Homo sapiens HG18 Expression Array (12x135k)], EGFR expression was assessed using the following 16 probe sets: BC094761P02842, BC094761P03082, BC094761P03230, K03193P01468, K03193P01728, NM_005228P04327, NM_005228P04420, NM_005228P04740, NM_201283P01192, NM_201284P01499, NM_201282P02053, NM_201283P01046, NM_201282P01192, NM_201284P01366, NM_201282P01499, and NM_201284P01555; and STAT3 expression was assessed using the following 12 probe sets: BC000627P01545, BC000627P02286, BC000627P02463, BC014482P02284, BC014482P02461, BC094761P03082, NM_003150P03804, NM_003150P04056, NM_003150P04263, NM_213662P03671, NM_213662P03923, and NM_213662P04130. In GSE39388 (based on Affymetrix Human Genome U133 Plus 2.0 Array), EGFR expression was assessed using the following 7 probe sets: 1565483_at, 1565484_x_at, 201983_s_at, 201984_s_at, 210984_x_at, 211607_x_at, and 224999_at; and STAT3 expression was assessed using the following 3 probe sets: 208991_at, 208992_s_at, and 225289_at. Relative EGFR/STAT3 expression was obtained by normalizing the expression levels from each cell model to the average of the expression levels obtained in control cells.

### 2.5 *In silico* analysis of RNAseq and protein data from TCGA‐PRAD

The level‐3 molecular data from The Cancer Genome Atlas Prostate‐Adenocarcinoma (TCGA‐PRAD), namely the RNA and protein expression available for 497 and 351 tumors, respectively, were retrieved from the UCSC Xena browser [38]. The tumors were subtyped as ETS negative, ETV1 positive, or ERG positive according to the log2 normalized expression of ETS transcripts (ETV1, ETV4, ETV5, ERG and FLI1). “ETV1 positive” were defined as having ETV1 expression within the highest 30% and ERG expression within the lowest 50% (*n* = 62). “ERG positive” were defined as having ERG expression within the highest 50% and ETV1, ETV4, and ETV5 expression within the lowest 70% (*n* = 142). “ETS negative” were defined as having ETV1, ETV4, and ETV5 expression within the lowest 70% and ERG expression within the lowest 50% (*n* = 109) (Fig. S1A–C). These cut‐off expression levels were selected considering the overall described frequencies of ETS rearrangements/overexpression in PCa and their mutual exclusivity.

### 2.6 Assessment of drugs' IC50s

The EGFR inhibitor Erlotinib (Selleck Chemicals, Berlin, Germany) and the STAT3 inhibitor TTI‐101 (Selleck Chemicals) were diluted in DMSO, and treatments were performed for 48 h, with appropriate DMSO‐matched controls (Table S1). Serial dilutions of Erlotinib and TTI‐101 were used in PNT2 and LNCaP wild‐type cells to determine the concentration that leads to ~ 50% decrease in cell viability (IC50). Erlotinib was tested at concentrations of 0.1, 0.5, 5, 10, 20, 50, and 100 μm in LNCaP cells and at concentrations of 0.1, 0.5, 1, 2, 3, 4, 5, 7.5, 10, 12.5, 15, 20, 25, 30, 40, 50, and 100 μm in PNT2 cells. TTI‐101 was tested at concentrations of 5, 10, 20, 30, 40, and 50 μm for both PNT2 and LNCaP cells. Cell viability was quantified using the MTT (3‐(4,5‐dimethylthiazol‐2‐yl)‐2,5‐diphenyltetrazolium bromide; Sigma‐Aldrich) assay. Briefly, PNT2 and LNCaP cells were seeded in 96‐well plates (Falcon, Corning, NY, USA) at 3.0 × 10^4^ and 4.0 × 10^4^ cells per well, respectively. Cell viability was measured immediately prior to drug treatments (T0) and 48 h postdrug treatment (T48). At each time point, 50 μL of MTT (0.5 mg·mL^−1^ in cell growth medium) was added to each well, followed by a 2‐h incubation at regular growth conditions. After discarding the solution, formazan crystals were solubilized with 100 μL DMSO, and absorbance was read at 570 nm using a microplate reader (Fluostar Omega, BMG Labtech, Ortenberg, Germany), with background correction at 550 nm. DMSO only was used for blank correction. The IC50 value for each drug was determined using the GRcalculator [39] using dose–response measurements normalized to DMSO‐treated controls. A Traditional Sigmoidal Normal model curve fitted to the relative cell viability was used to define IC50 values.

### 2.7 2D high‐throughput cell viability and apoptosis assay

We established a high‐throughput method to quantify cell viability and apoptosis in cells seeded in 384‐well plates using dual staining with Calcein AM and Propidium Iodide (PI), respectively. Briefly, PNT2 wild‐type cells were seeded at 2.0 × 10^3^ cells per well, and after cell adherence, growth medium was removed and different concentrations of Calcein AM (0.1, 1, 2 and 4 μm in PBS) and PI [0.16%, 0.2%, 0.4% and 0.8% (v/v) in PBS] were added to cells' growing as a 2D monolayer. Cells were incubated for 20 min at regular growth conditions protected from light. Excess staining solution was removed, and cells were rinsed twice with PBS (1×). Stained cells were imaged using the Cytation C10 Confocal Image Reader (Agilent BioTek, Santa Clara, CA, USA) with fluorescence filters for Calcein AM (GFP filter: 488/509 nm) and PI (Texas Red filter: 561/617 nm). Microscope settings used included: Threshold 4000 RIU, Dark background, Split Touching Objects, Fill holes in object masks, Auto Background Flattening Size (300 μm), Background Percentage: 5%, Minimum Object Size: 5 μm, Maximum Object Size: 100 μm, Plug Shape: Square/Rectangle, Plug Size X: 2289 μm, Plug Size Y: 2126 μm, Plug X Offset: −239 μm, Plug Y Offset: 106 μm. *Gen5* software (Agilent BioTek) was used to quantify live (Calcein AM‐positive) and apoptotic (PI‐positive) cells using predefined algorithms. Brightness and contrast of images were adjusted using fiji software (Max Planck Institute of Molecular Cell Biology and Genetics, Dresden, Germany). Optimization experiments revealed that 0.1 μm Calcein AM was optimal for staining prostate cell nuclei, providing well‐defined cellular markers without cytoplasmic dispersion (Fig. S2A). High‐throughput screening (HTS) using automated object identification was employed to provide robust, unbiased quantifications of Calcein AM‐ (Fig. S2B,C) and PI‐ (Fig. S2D,E) positive cells.

### 2.8 Assessment of drugs' synergism

Following the MTT protocol detailed above, PNT2 and LNCaP wild‐type cells were seeded in 96‐well plates and cotreated with different combinations of Erlotinib (1.0, 5.0, and 10 μm) and TTI‐101 (1, 2.5, 5, 10, 25, and 50 μm), or DMSO (as controls), for 48 h. Similarly, LNCaP and VCaP wild‐type cells were seeded into 384‐well plates at 2.0 × 10^3^ cells per well. Brightfield images were acquired in the Cytation C10 Confocal Image Reader before (T0) and after (T48) treatment. Cell quantifications were performed using *Gen5* software (Agilent BioTek) and drug synergism was calculated using the SynergyFinder+ Web Application [40]. The Highest Single Agent (HSA) synergistic model was employed to calculate synergy scores. This model assumes that a combination exhibits synergy only if its effect surpasses the maximum effect of any single agent in the combination. Synergy scores were automatically calculated using cell viability percentage data for all drug combinations and monotherapy treatments, with values > 10 indicating synergy, < −10 indicating antagonism, and scores between −10 and 10 interpreted as additive interactions. Heatmaps were generated directly from the *software* to illustrate the interaction between Erlotinib and TTI‐101 across the tested concentrations. Monotherapy synergy scores (outputted as zero) were excluded from the heatmaps. The experiment was performed twice, and in each experiment, four wells were included per condition.

### 2.9 Coinhibition of EGFR and STAT3 in 2D and 3D spheroid models

For 2D models, PNT2 cells were seeded in 384‐well plates at 2.0 × 10^3^ cells per well and, after adherence, cells were treated with 10 μm TTI‐101 + 5 μm Erlotinib or 0.1% DMSO (controls) for 48 h. Three independent experiments were performed and, in each experiment, eight wells were included per condition. The growth rate (GR) was calculated using the natural logarithm of the ratio of the change in viable cell count over time in treated and control conditions, normalized to the initial cell count difference between treated and control conditions [41]. The apoptotic fraction was determined by calculating the percentage of apoptotic cells relative to the total cell count in each condition.

For 3D spheroid models of LNCaP and VCaP cells, we optimized the number of cells that allowed us to achieve full spheroid maturation using ultra‐low attachment 384‐well plates (Corning, NY, USA). We observed that both LNCaP and VCaP spheroids maintain structural integrity over time, with 2500 and 2000 cells per well, respectively, selected as the ideal cell seeding density. LNCaP spheroids formed irregular shapes with a mean area of 1.07 × 10^5^ μm^2^ and a mean diameter of 3.74 × 10^2^ μm at T0 (96h postseeding) (Fig. S3A). In contrast, VCaP spheroids, despite being smaller, were more circular, compact, and homogeneous at the same time point, with a mean area of 2.92 × 10^4^ μm^2^ and a mean diameter of 1.92 × 10^2^ μm (Fig. S3B).

After seeding, cells were centrifuged at 300 ***g*** for 1 min to accelerate spheroid formation. Brightfield images were acquired every 24 h for 8 days to monitor spheroid development. Cell culture medium was changed every 48 h, after a centrifugation step (300 ***g***, 1 min) to minimize spheroid loss. Following spheroid formation, cells were cotreated with 10 μm TTI‐101 + 5 μm Erlotinib or 0.1% DMSO (controls) for 96 h. Brightfield images with 10 z‐stacks (10–13 μm slices) were acquired before treatment (T0) and every 24 h (T24, T48, T96). Image analysis was performed on z‐projected images using *Gen5* software (Agilent BioTek) and measurements included spheroid size, area, circularity, and perimeter. At least three independent assays were performed for each cell line.

### 2.10 Statistical analyses

For quantification of western blots and for microarray expression data obtained from the GEO DataSets database, the unpaired *t*‐test was used individually to determine statistically significant differences between samples. For TCGA‐PRAD data, the Shapiro–Wilk normality test was used to assess variable normality. The gene‐level transcription estimates, as the log_2_(*x* + 1)‐transformed RSEM normalized counts, and the normalized relative levels of protein expression were visualized as box‐plots. Statistically significant differences between two groups were evaluated using the nonparametric Wilcoxon rank‐sum test, while the Kruskal–Wallis test was used to compare multiple groups. For drug synergism studies, SynergyFinder+ employs a resampling‐based method to generate *P*‐values for synergy scores [40]. For drug combination experiments, in 2D models, the paired *t*‐test was used, while in 3D models, the multiple paired *t*‐test was used. Where applicable, n.s. = *P* > 0.05, * = *P* < 0.05, ** = *P* < 0.01, *** = *P* < 0.001, and **** = *P* < 0.0001.

## 3 Results

### 3.1 EGFR expression and phosphorylation at Tyr1068 are differentially regulated by ETV1 and ERG overexpression

**Fig. 1:**
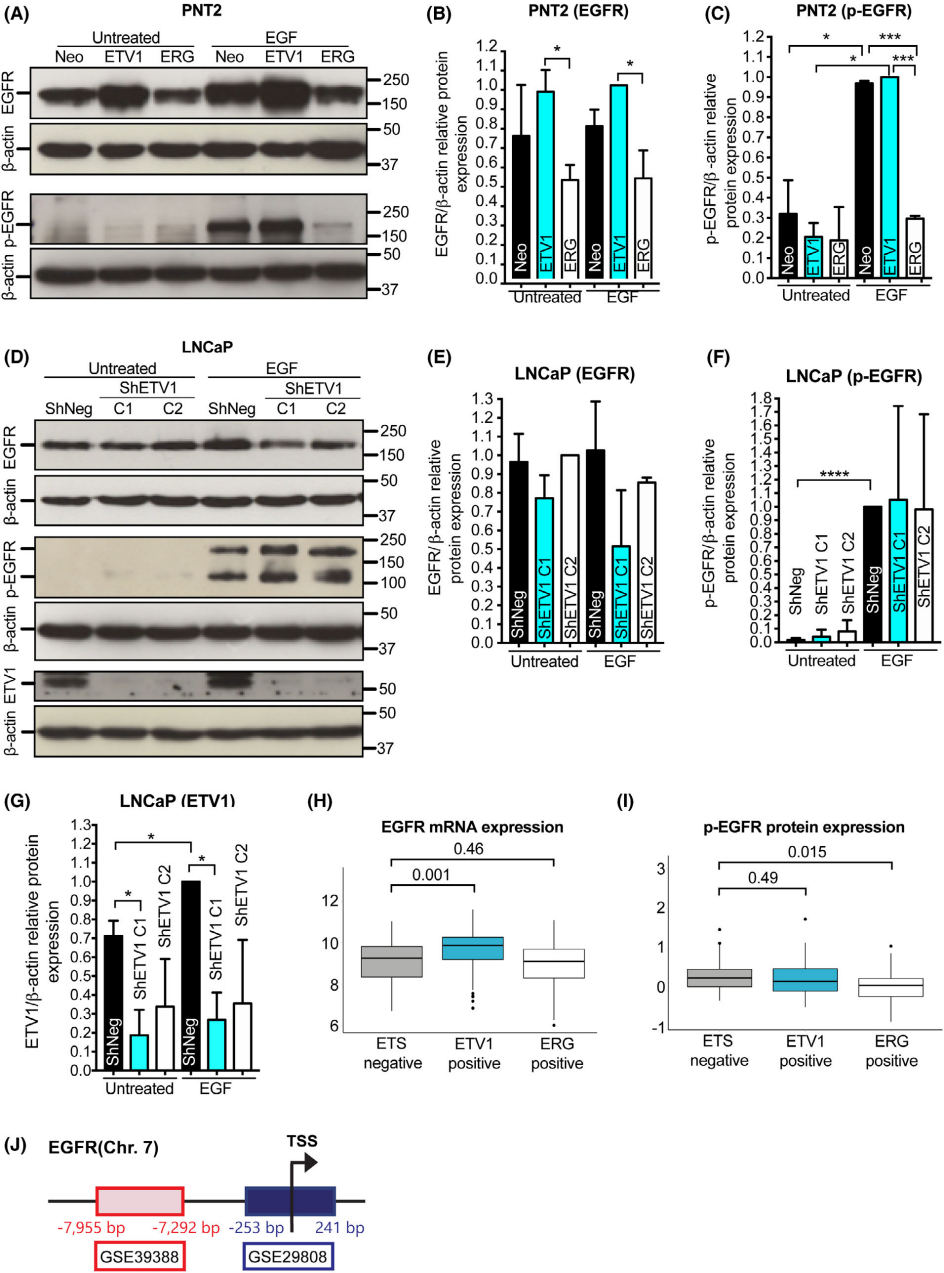
EGFR expression and activity is modulated by ETS overexpression in prostate cell models and tumors. (A) Representative protein blot probed to reveal EGFR and phospho‐EGFR (p‐EGFR, Tyr1068) expression in PNT2 control (Neo) and PNT2‐derived cells overexpressing ETV1 (ETV1) or ERG (ERG), upon exposure or not to EGF stimuli (EGF vs. Untreated, respectively). β‐Actin was used as a loading control. (B, C) Quantification of the relative protein expression of EGFR (B) or p‐EGFR (C) in PNT2‐derived cell populations. Relative EGFR/p‐EGFR expression to β‐Actin in each cell population was normalized to the sample showing the highest expression. (D) Representative protein blot probed to reveal EGFR, p‐EGFR, and ETV1 expression in LNCaP control (ShNeg) and LNCaP‐derived cells depleted of ETV1 (ShETV1 C1 and C2), upon exposure or not to EGF stimuli (EGF vs. Untreated, respectively). β‐Actin was used as a loading control. (E, F) Quantification of the relative protein expression of EGFR (E), p‐EGFR (F) or ETV1 (G) in LNCaP‐derived cell populations. Relative EGFR/p‐EGFR/ETV1 expression to β‐Actin in each cell population was normalized to the sample showing the highest expression. (H, I) Analysis of EGFR mRNA expression (H) and p‐EGFR protein expression (I), in “ETS‐negative” (*n* = 109), “ETV1‐positive” (*n* = 62), and “ERG‐positive” (*n* = 142) tumors obtained from the TCGA‐PRAD dataset; *y*‐axis indicates normalized relative expression of EGFR mRNA and p‐EGFR protein. (J) Schematic representation of genomic regions obtained from ChiP‐seq for the EGFR gene, where TSS represents the Transcription Start Site and red and blue rectangles represent the ETV1‐bound genomic regions identified from GSE39388 and GSE29808 data, respectively. In A and D, numbers on the right side of the blots indicate the position of the protein markers (kDa). Two independent experiments were performed for the PNT2 model (*n* = 2) and three for the LNCaP model (*n* = 3). In B, C, E, F, and G, the unpaired *t*‐test was used to determine statistically significant differences between samples, while in H and I, the Kruskal–Wallis test was used. For simplification, whenever *P* < 0.05 the following symbol code was used: * for *P* < 0.05, ** for *P* < 0.01, *** for *P* < 0.001, and **** for *P* < 0.0001. Error bars represent standard deviation.

To determine whether ETV1 expression affects the expression and activation of EGFR in prostate cells, we quantified the levels of total EGFR and Tyr1068‐phosphorylated EGFR (p‐EGFR) upon stimulation with EGF in two prostate cell lines with modulation of ETV1 expression: the nontumorigenic PNT2 cells with *de novo* ETV1 overexpression, mimicking early‐stage PCa development, and the tumorigenic LNCaP cells with stable silencing of ETV1 expression, mimicking advanced PCa with and without ETV1 overexpression (Fig. 1). In PNT2 cells, we observed that *de novo* overexpression of ETV1 leads to increased EGFR expression independently of EGFR stimulation, while ERG overexpression does not enhance EGFR expression under the same conditions (Fig. 1A,B). In agreement, upon stimulation with EGF, EGFR activation (p‐EGFR) is significantly increased in *de novo* ETV1 overexpressing cells, but not in ERG‐overexpressing cells (Fig. 1A,C). On the other hand, *de novo* ERG overexpression appears to repress EGFR phosphorylation in EGF‐treated cells (Fig. 1A,C).

In tumorigenic LNCaP cells, silencing of ETV1 led to a slight decrease in total EGFR expression, independently of EGFR stimulation (Fig. 1D,E), supporting the findings in PNT2‐ETV1 cells and suggesting that EGFR expression is regulated, at least in part, by ETV1. ETV1 silencing, however, was not sufficient to decrease EGFR phosphorylation at Tyr1068 (Fig. 1D,F).

These associations were also observed in publicly available data from studies using RWPE‐1, LNCaP, and VCaP cells (namely, GSE29438 and GSE39388) with modulation of ETV1 and/or ERG expression, where *de novo* expression of *ETV1* in RWPE‐1 cells, but not of *ERG*, was sufficient to induce a significant increase in *EGFR*; however, the silencing of *ETV1* or *ERG* in the tumorigenic LNCaP and VCaP cells, respectively, did not impact *EGFR* expression (Fig. S4A–D). Additionally, *in silico* analysis of publicly available ChIP‐seq data from two studies using different prostate cell lines with modulation of ETV1 or ERG expression (GSE39388 and GSE29808) identified EGFR among the list of ETV1 targets only, with both studies demonstrating direct ETV1 binding to the promoter region of EGFR (Fig. 1J).

Since the EGFR pathway may activate downstream transcription factors as STATs, we questioned whether EGFR activation could also modulate ETV1 expression. For this purpose, we analyzed ETV1 protein expression levels in LNCaP‐derived cells that were either untreated or stimulated with EGF. In control cells (ShNeg), we observed that ETV1 expression was significantly increased by ~ 1.4‐fold upon EGFR stimulation (Fig. 1D,G). The same tendency was observed in PNT2‐derived cells, with EGF stimulation leading to increased ETV1 expression, but not increased ERG, in all cell models (Fig. S5A). Conversely, in the ERG‐overexpressing VCaP cells, EGF treatment did not show to consistently impact ERG or p‐EGFR expression, and a tendency for decreased EGFR was observed (Fig. S5B), in line with that observed in PNT2‐ERG cells. Collectively, these results suggest a positive feedback loop between EGFR and ETV1, with ETV1 inducing EGFR activation, and EGF stimuli increasing both EGFR and ETV1 expression. In agreement with this, data obtained for the Prostate Adenocarcinoma tumor samples from The Cancer Genome Atlas (TCGA‐PRAD) showed a significant increase in EGFR expression and a tendency for increased p‐EGFR expression in ETV1‐positive PCa (Fig. 1H,I), supporting differential EGFR regulation by ETS proteins and a positive association between EGFR and ETV1 expression in human PCas.

Taken together, these results place EGFR as a mediator of ETV1 oncogenic activity both in early and advanced prostate carcinogenesis, while ERG overexpression seems to indirectly repress EGFR activation in early PCa carcinogenesis.

### 3.2 STAT3 and STAT5A are downstream effectors of ETV1 oncogenic signaling

**Fig. 2:**
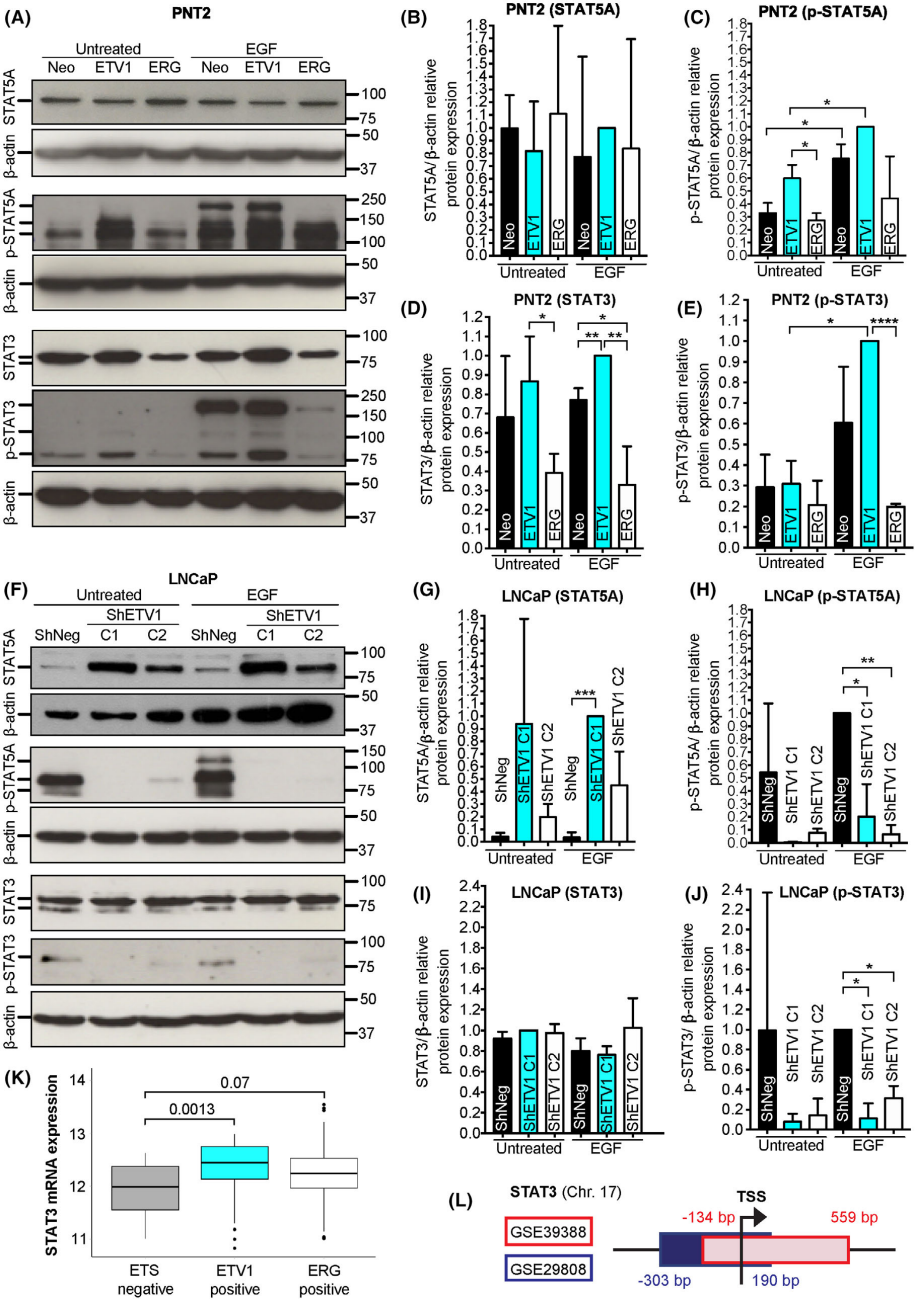
ETV1 Overexpression activates STAT3 and STAT5A. (A) Representative protein blot probed to reveal STAT5A, phospho‐STAT5A (p‐STAT5A, Tyr694), STAT3 and phospho‐STAT3 (p‐STAT3, Tyr705) in PNT2 control (Neo) and PNT2‐derived cells overexpressing ETV1 (ETV1) or ERG (ERG), upon exposure or not to EGF stimuli (EGF vs. Untreated, respectively). β‐actin was used as a loading control. (B–E) Quantification of STAT5A (B), p‐STAT5A (C), STAT3 (D) and p‐STAT3 (E) relative protein expression in PNT2‐derived cell populations. Relative STAT/p‐STAT expression to β‐actin in each cell population was normalized to the sample showing the highest expression. (F) Representative protein blot probed to reveal STAT5A, p‐STAT5A, STAT3, and p‐STAT3 in LNCaP control (ShNeg) and LNCaP‐derived cells depleted of ETV1 (ShETV1 C1 and C2), upon exposure or not to EGF stimuli (EGF vs. Untreated, respectively). β‐actin was used as a loading control. Numbers on the right side of the blots indicate molecular weights (kDa). (G–J) Quantification of STAT5A (G), p‐STAT5A (H), STAT3 (I) and p‐STAT3 (J) relative protein expression in LNCaP‐derived cell populations. Relative STAT/p‐STAT expression to β‐Actin in each cell population was normalized to the sample showing the highest expression. (K) Analysis of STAT3 mRNA expression in “ETS negative” (*n* = 109), “ETV1 positive” (*n* = 62) and “ERG positive” (*n* = 142) tumors obtained from the TCGA‐PRAD data collection; *y*‐axis indicates normalized relative expression for STAT3 mRNA levels. (L) Schematic representation of genomic regions obtained from ChiP‐seq for the STAT3 gene, where TSS represents the Transcription Start Site, and red and blue rectangles represent the ETV1‐bound genomic regions identified from GSE39388 and GSE29808 data, respectively. In A and F, numbers on the right side of the blots indicate the position of the protein markers (kDa). Two independent experiments were performed for the PNT2 model (*n* = 2) and three for the LNCaP model (*n* = 3). In B, C, D, E, G, H, I, and J, the unpaired *t*‐test was used to determine statistically significant differences between samples, while in K, the Kruskal–Wallis test was used. For simplification, whenever *P* < 0.05 the following symbol code was used: * for *P* < 0.05, ** for *P* < 0.01, *** for *P* < 0.001, and **** for *P* < 0.0001. Error bars represent standard deviation.

Building upon our findings demonstrating a positive correlation between ETV1 and EGFR expression in prostate cells, we further investigated the impact of ETV1 overexpression in EGFR downstream STATs signaling. For that purpose, we assessed the expression and activation of STAT3 and STAT5A in both PNT2‐derived and LNCaP‐derived cell lines with and without stimulation with EGF. In PNT2 cells, we observed increased levels of phospho‐STAT5A (p‐STAT5A, Tyr694), but not total STAT5A, in cells overexpressing ETV1 compared either to control cells or to ERG‐overexpressing cells, independently of EGF stimuli (Fig. 2A–C). On the other hand, both expression and activation of STAT3 (p‐STAT3, Tyr705) were significantly increased in ETV1 overexpressing cells after stimulation with EGF, with overexpression of ETV1, by itself, not being sufficient to significantly increase STAT3 expression (Fig. 2A,D,E). In contrast, ERG overexpression led to decreased expression and activation of STAT3, independently of EGF stimuli. These observations place STAT3 and STAT5A as downstream effectors of early carcinogenesis mediated by ETV1‐EGFR oncogenic signaling. mRNA expression data obtained from *de novo* ERG or ETV1 expression in RWPE‐1 cells did not show a consistent impact on STAT3 expression (Fig. S4E,F).

In the tumorigenic LNCaP cells, ETV1 depletion caused a dramatic decrease in both p‐STAT5A and p‐STAT3 independently of EGF stimuli, despite the increase observed in total STAT5A (Fig. 2F–J). In agreement, analysis of STAT3 expression in PCa tumors from the TCGA‐PRAD dataset revealed significantly higher levels of STAT3 mRNA in ETV1‐positive tumors compared to ETS‐negative tumors, with ERG‐positive tumors showing a tendency for increased STAT3 expression (Fig. 2K). Curiously, according to mRNA expression data from the GEO DataSets GSE29438 and GSE39388, in the tumorigenic LNCaP and VCaP cells, silencing of either ETV1 or ERG, respectively, led to a significant decrease in STAT3 mRNA expression (Fig. S4G,H). Regarding protein level, the TCGA‐PRAD dataset has only data for total STAT5A and p‐STAT3, for which no association was observed with ETS‐status (Fig. S1D,E). Nevertheless, by integrating ChIP data from GEO DataSets, we further reinforce the association between ETV1 and STAT3 expression, as STAT3 regulatory elements covering the TSS were found in two independent ChIP‐seq studies for ETV1‐linked chromatin, but not for ERG (Fig. 2L). Thus, altogether, these observations suggest that while STAT3 may be a direct target of ETV1, positively regulated by it in both early and advanced PCa cell models, STAT3 can also be (indirectly) regulated by ERG in advanced PCa. STAT5A was not found among the list of ETV1 or ERG downstream targets, suggesting indirect regulation by both transcription factors.

These results provide mechanistic insights into the role of ETV1 in activating STAT3 and STAT5A signaling in PCa cells, and support the rationale for targeted inhibition of STAT3/STAT5A for the treatment of ETV1‐overexpressing PCas.

### 3.3 Both ETV1 and ERG overexpression elicit sensitivity to EGFR and/or STAT3 inhibition in early prostate carcinogenesis

**Fig. 3:**
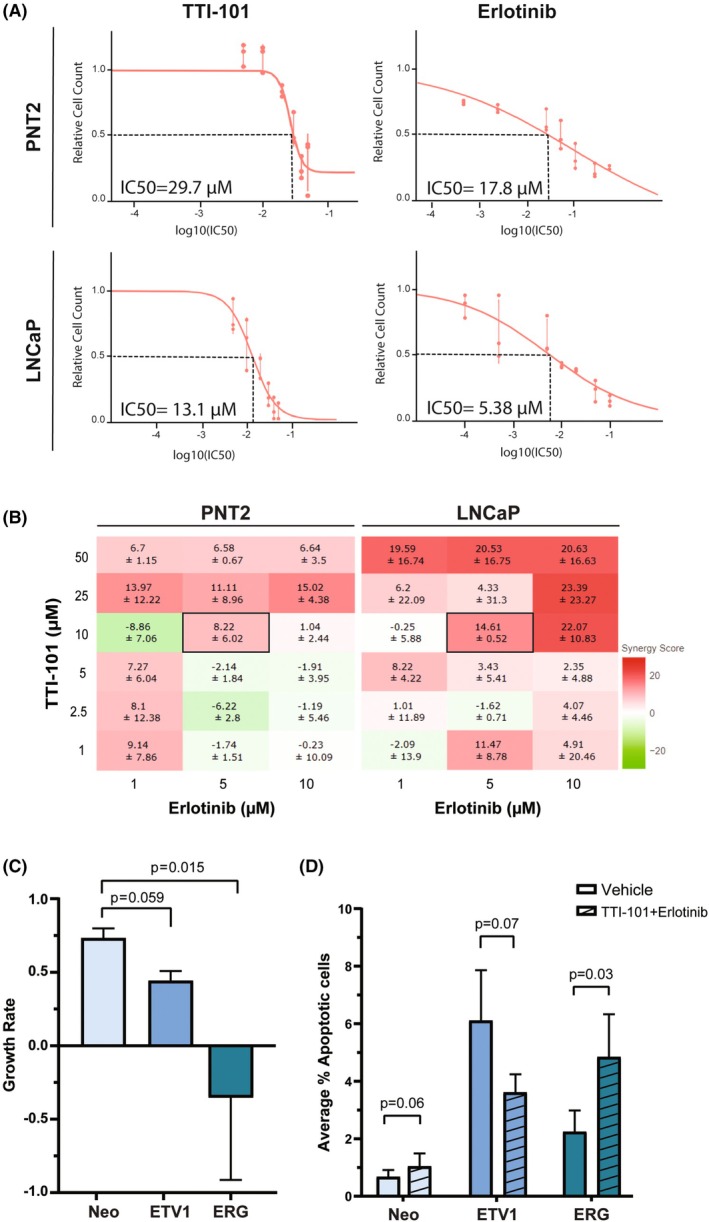
Coinhibition of EGFR/STAT3 with TTI‐101 and Erlotinib elicits a synergistic effect in ETS‐overexpressing prostate cancer cells. (A) IC50 concentrations obtained from the analysis of cell viability using the MTT assay in PNT2 and LNCaP cells treated with different concentrations of TTI‐101 (STAT3 inhibitor) or Erlotinib (EGFR inhibitor) for 48 h. IC50 values were determined using the GR online calculator [39]. Error bars represent the standard deviation from three independent experiments. (B) Heat maps outputted by the SynergyFinder+ Web Application, representing the impact on cell viability imposed by treatments with different dose combinations of TTI‐101 and Erlotinib in 2D PNT2 and LNCaP cell models in 96‐well plates. Primary data was obtained using the MTT assay. For each drug combination, a synergy score is shown: negative values (pointing to antagonistic effect) are shown in green colors, and positive values (pointing to synergistic effect) are shown in red colors. Note that monotherapy values are not shown since they present a synergy score of zero. Dark squared values highlight the selected dose combination used in further experiments (10 μm TTI‐101 + 5 μm Erlotinib). Data represent mean ± SD from two independent experiments. (C, D) Quantification of growth rates, obtained with the MTT assay (C), and of the average percentage of apoptotic cells (D), in PNT2 control (Neo) and PNT2‐derived cells overexpressing ETV1 or ERG, upon cotreatment with TTI‐101 + Erlotinib at the defined concentrations (5 and 10 μm, respectively) for 48 h. To assess statistical significance of the differences between samples (C) or between treatment schemes (D), the paired *t*‐test was used. Error bars represent standard deviation from three independent experiments.

To explore the sensitivity of prostate cells to EGFR and STAT3 inhibitors, we began by determining the IC50 concentrations for the EGFR inhibitor Erlotinib and the STAT3 inhibitor TTI‐101 in the wild‐type nontumorigenic PNT2 and in the wild‐type tumorigenic LNCaP cell lines. We observed that the IC50 values for both Erlotinib and TTI‐101 were higher in PNT2 cells (17.8 and 29.7 μm, respectively) than in LNCaP cells (5.4 and 13.1 μm, respectively) (Fig. 3A), suggesting that PCa cells are more sensitive to EGFR or STAT3 inhibition than nontumorigenic prostate cells. When comparing both drugs, Erlotinib demonstrated greater potency than TTI‐101 in reducing cell viability, with lower IC50 values observed for each cell line (Fig. 3A). Interestingly, in the tumorigenic PCa cells 22Rv1, which exhibit an ETS profile similar to that of PNT2 cells [10], the obtained IC50 values were even greater than those obtained for LNCaP cells – 66.4 and 31.7 μm for Erlotinib and TTI‐101, respectively (Fig. S6), supporting the influence of the ETS cell context, rather than a general feature of cancer cells.

To determine the efficacy of coinhibition of EGFR and STAT3, we cotreated 2D cultures of the nontumorigenic PNT2 and tumorigenic LNCaP cells with varying concentrations of TTI‐101 and Erlotinib and assessed cell viability. Using heat maps to visualize the effects of different dose combinations, we observed increased sensitivity to the combined treatments compared to single‐agent treatments (Fig. 3B), in accordance with the synergistic effect of both inhibitors. Specifically, the combination of 10 μm TTI‐101 with 5 μm Erlotinib exhibited consistently higher synergistic efficacy in the tumorigenic LNCaP cells compared with the nontumorigenic PNT2 cells (synergy score of 14.61 ± 0.52 versus 8.22 ± 6.02, respectively; Fig. 3B). The same output was obtained using the *combenefit* software (Cancer Research UK Cambridge Institute, University of Cambridge, Cambridge, UK) (Fig. S7A).

Then, to determine whether oncogenic ETV1 overexpression could underlay synergic inhibition with TTI‐101 and Erlotinib, we explored the potential cytotoxic impact of the most effective drug combination (10 μm TTI‐101 with 5 μm Erlotinib) in PNT2‐derived cell populations with *de novo* ETV1 or ERG overexpression, using dual staining using Calcein AM and Propidium Iodide (PI) to assess cell viability and apoptosis, respectively. After 48 h of combined treatment with 10 μm TTI‐101 + 5 μm Erlotinib, we observed a noticeable decrease in growth rate (~ 60%) in ETV1‐overexpressing cells when compared to PNT2 Neo controls (~ 25%), and a complete loss of cell viability in ERG‐overexpressing cells (Fig. 3C; Fig. S8). In the same cell populations, a concomitant significant increase in apoptosis was observed in ERG‐overexpressing cells upon treatment with TTI‐101 + Erlotinib, but not in control (Neo) or ETV1‐overexpressing cells (Fig. 3D; Fig. S8).

Although activation of the EGFR‐STAT3 pathway was found to be increased in ETV1‐positive PNT2‐derived cells only, these results suggest that in early tumorigenesis, both ETV1‐ or ERG‐mediated oncogenic signaling may exhibit sensitivity to the combination of the inhibitors Erlotinib and TTI‐101, despite the affected signaling cascade being different.

### 3.4 Synergistic activity of TTI‐101 and erlotinib consistently impairs growth of spheroid models of advanced ETV1‐positive prostate cancer

**Fig. 4:**
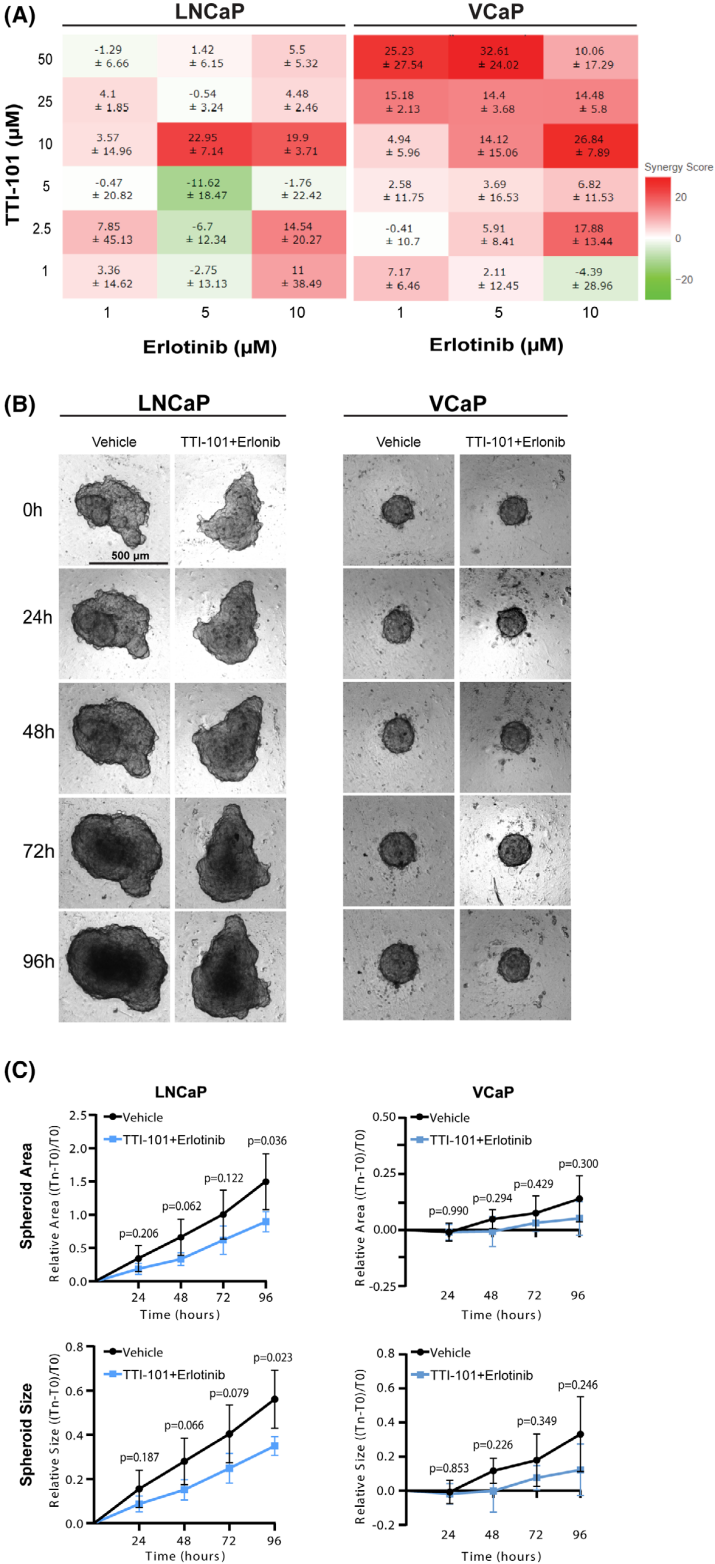
Combined TTI‐101 and Erlotinib treatment shows synergistic effects in spheroid models of advanced ETV1‐positive prostate cancer. (A) Heat maps outputted by the SynergyFinder+ Web Application, representing cell viability with different combinations of drug treatments using TTI‐101 and Erlotinib in 2D LNCaP and VCaP cell models, in 384‐well plates. Primary data was obtained using the Calcein AM/Propidium Iodide cell viability and apoptosis assay in a 384‐well plate format. Note that monotherapy values are not shown since they present a synergy score of zero. Three independent experiments were performed. (B) Representative brightfield images of LNCaP and VCaP 3D spheroids treated with vehicle (0.1% DMSO) or with TTI‐101 + Erlotinib (10 μm + 5 μm, respectively) at T0 (4 days after cell seeding), and imaged every 24 h. Three independent experiments were performed. Scale bar = 500 μm. (C) Quantification of LNCaP (left) and VCaP (right) spheroids' area and size upon treatment with vehicle (0.1% DMSO) or TTI‐101 + Erlotinib at 24 h intervals, up to 96 h of treatment. To assess statistical significance of the differences between treatment schemes at each time point, the multiple paired *t*‐test was used. Error bars represent standard deviation from three independent experiments.

To test whether the previously observed effects were also observed in cell models of advanced PCa with underlying ETV1 or ERG overexpression, we compared the effects of EGFR and STAT3 coinhibition in LNCaP and VCaP cells, respectively. Synergism was evaluated in both cell models using Calcein‐AM staining. A strong synergism was consistently observed using 10 μm TTI‐101 with 5 μm Erlotinib in LNCaP cells (synergy score of 22.95 ± 7.14), while VCaP cells showed lower sensitivity to this combination scheme (synergy score of 14.12 ± 15.06), with higher concentrations of Erlotinib (10 μm) being required to achieve an equivalent synergistic effect (Fig. 4A). A similar output was obtained using the *combenefit* software (Fig. S7B).

To evaluate whether the synergistic interaction between TTI‐101 and Erlotinib is maintained in more complex cellular models, we established 3D cultures of both LNCaP and VCaP cells and monitored spheroid size and area as a function of time after treatment. Analysis of spheroid growth revealed that coinhibition of EGFR and STAT3 leads to a statistically significant decrease in the size and area of LNCaP spheroids at T96 (96 h of treatment) (Fig. 4B,C). This effect was already evident at T48 and T72, showing borderline significance compared to controls. Combined treatment with TTI‐101 and Erlotinib at the defined dose combination showed no significant impact on VCaP spheroid size or area. In fact, in VCaP cells, high heterogeneity was observed within and between assays, despite the well‐structured shape of the formed spheroids (Fig. S3C,D). These findings support that ETV1‐overexpressing PCa cells may be more sensitive to the synergistic interaction between TTI‐101 and Erlotinib and sustain the potential of cotargeting EGFR and STAT3 pathways as a therapeutic strategy in advanced prostate carcinomas displaying ETV1 overexpression.

## 4 Discussion

This study elucidates the intricate interplay between ETS rearrangements and the EGFR‐STATs signaling pathway in PCa cells and underscores the potential of a personalized targeted therapy for the molecular subgroup of prostate carcinomas characterized by ETV1 overexpression, which represents ~ 10% of all PCa patients and is associated with a worse prognosis. The use of single pharmacological agents is common in cancer treatment, but combination therapy using two or more pharmacological strategies has garnered significant attention in recent years through the promise of enhancing drug efficacy, reducing toxicity, and targeting multiple cancer mechanisms [42]. However, studying the complexity of multiple drugs and treatment schemes can be challenging.

Aiming to identify targets that may potentiate the clinical utility of an anti‐EGFR therapy, in this work, we explored EGFR as an ETV1‐associated targetable receptor and dissected STAT3 and STAT5A as downstream effectors of ETV1‐EGFR signaling. Taking advantage of high‐throughput screening approaches and using models of PCa cells mimicking early (PNT2‐ETV1 and PNT2‐ERG) and advanced (LNCaP and VCaP) prostate cancer, we gained better insights into the relevance of the EGFR/STATs pathway for ETS‐driven prostate carcinogenesis. In fact, while the established PNT2‐derived cells with *de novo* ETV1 or ERG overexpression constitute clear cell models to identify ETS‐driven oncogenic pathways, with ETS overexpression being introduced as the single driver of the acquisition of a malignant phenotype [10, 43, 44], the cell models LNCaP and VCaP, with underlying *ETV1* and *ERG* rearrangements/overexpression, respectively, constitute ETS‐driven models of advanced PCa, providing insightful validation of the therapeutic potential of targeted approaches.

We have identified, for the first time, a positive feedback loop between EGFR activation and ETV1 expression, under which ETV1 overexpression leads to increased EGFR levels and activity, and EGF stimulation leads to increased ETV1 protein levels, creating a cycle that promotes tumorigenic signaling. This positive feedback was observed in both early (PNT2‐ETV1 cells) and advanced (LNCaP cells) models of ETV1‐driven oncogenic activity, while a link with ERG was not observed in the corresponding ERG‐positive cell models (PNT2‐ERG and VCaP cells), sustaining the potential efficacy of EGFR inhibitors for the treatment of ETV1‐positive prostate carcinomas.

Aiming to identify a combination therapy for this molecular subtype, we explored EGFR activation of downstream STATs as possible targets of the ETV1‐EGFR oncogenic axis. For that purpose, we started by exploring the ETS specificity of EGF‐activated EGFR/STATs in PNT2‐derived cell populations. Assessing the activity and expression levels of STAT3 and STAT5A by western blot, both proteins showed to be positively regulated by *de novo* ETV1 overexpression, while *de novo* ERG overexpression showed an inverse effect, with ETV1 leading to increased levels of pSTAT5A and p‐STAT3, and ERG overexpression leading to lower levels of both pSTAT5A and p‐STAT3. This link with ETV1 was maintained in the advanced ETV1‐positive PCa cells LNCaP, where ETV1 depletion led to decreased p‐STAT5A and p‐STAT3. The differential ETS regulation had, actually, been observed for p‐EGFR in the same cell models, despite ETV1 silencing in LNCaP cells not being sufficient to decrease p‐EGFR levels. To get further insights into the mechanisms underlying the EGFR/STATs ETS regulation and to expand the relevance of our findings, we explored external data available from both GEO DataSets and TCGA‐PRAD. Interestingly, ChIP‐seq data from two different studies unveiled both EGFR and STAT3, but not STAT5A, as direct downstream targets of ETV1, but not of ERG, in line with the observed impact of ETV1 overexpression in PNT2‐derived cells and of ETV1 silencing in LNCaP cells. Additionally, ETV1‐positive PCa samples from TCGA‐PRAD showed a statistically significant increase in both EGFR and STAT3 expression when comparing with ETS‐negative PCa, while STAT5A was not associated with the ETS profile. The fact that this relationship can be detected in TCGA‐PRAD tumor samples, expected to better represent the heterogeneity of each molecular subtype, strongly supports that the observed regulatory interaction between EGFR/STAT3 and ETV1 is likely to persist *in vivo*, independently of the tumor stage. In fact, although we have used stringent criteria to generate TCGA‐PRAD ETS groups with low ETS heterogeneity, considering that ETV1 and ERG overexpression are known to be mutually exclusive [3], the “ETV1‐positive” group is actually the one that exhibits some heterogeneity in the ETS profile, as overexpression of either ETV4 or ETV5 was not considered an exclusion criterium. Since ETV4 or ETV5 belong to the same subfamily as ETV1—the PEA3 subfamily—with known overlapping targeted pathways in the prostate [10], and co‐overexpression of ETV1 with ETV4 or ETV5 was common among TCGA‐PRAD samples, selecting “ETV1‐only” samples would result in a very short ETS group, without statistical power, which would not faithfully represent the ETV1‐positive subtype. Thus, the association between EGFR/STAT3 expression and the ETV1‐positive molecular subtype can be actually stronger, rather than weaker.

On the other hand, since silencing of ETV1 in LNCaP cells led to an increase in STAT5A expression, neither considered a direct ETV1 target nor found associated with the ETS profile in TCGA‐PRAD, it is possible that an ETV1 intermediate player, not overall specifically ETV1‐regulated, is repressing STAT5A expression in LNCaP cells, or that depletion of the ETV1 oncogene may lead to the accumulation of total STAT5A, which requires further investigation.

In light of the evidence pinpointing primed activation of the EGFR‐STAT3 pathway in PCa cells with ETV1 overexpression, we thus explored the antitumorigenic potential of a therapy combining Erlotinib and TTI‐101, as EGFR and STAT3 inhibitors, for the treatment of ETV1‐positive PCa. In cell models of early ETS‐driven PCa, we observed that coinhibition of EGFR and STAT3 pathways synergistically impaired cell growth in either ETV1‐ or ERG‐overexpressing cells, suggesting potential utility of the combination of TTI‐101 and Erlotinib for the treatment of both ETS‐positive PCa subtypes. Actually, this therapeutic approach was even more dramatic in PNT2‐ERG than in PNT2‐ETV1 cells. Although this requires further investigation, it is possible that, despite the low basal levels of EGFR/p‐EGFR and STAT3/p‐STAT3 in PNT2‐ERG cells (untreated cells in Figs 1 and 2), early‐oncogenic ERG signaling may be highly dependent on EGFR/STAT3 activation, and/or that Erlotinib and/or TTI‐101 are acting on other critical intracellular players of ERG‐mediated oncogenic signaling. In fact, Erlotinib has been reported to also inhibit HER2 in cells lacking EGFR [45], and although TTI‐101 is reported to target the phospho‐Tyr705‐peptide binding site within the SH2 domain of STAT3, potentially lacking “off‐targets”, studies of its impact on other STATs have not yet been reported. Nevertheless, the potential utility of the Erlotinib+TTI‐101 combination therapy for the treatment of ERG‐overexpressing PCa can broaden the first‐line therapeutic approaches currently available for early‐stage PCa, which requires further investigation.

To expand the validation of the potential efficacy of Erlotinib and TTI‐101 combination in advanced PCa, we performed synergistic analysis and growth impairment studies in 3D cell models of LNCaP and VCaP cells, as cell models of advanced ETV1‐ and ERG‐driven oncogenic signaling, respectively. However, consistent impairment of 3D cell growth was observed in LNCaP cells only. Despite the plethora of genetic and molecular specificities that may underlie LNCaP cells' response to this or any other therapeutic scheme, the consistency observed for both early and advanced ETV1‐overexpressing cell models (PNT2‐ETV1 and LNCaP cells, respectively) regarding activation of EGFR and STAT3, as well as their effective response to the corresponding inhibitors Erlotinib and TTI‐101, respectively, sustains the potential of combining these inhibitors to efficiently target prostate carcinomas with ETV1 overexpression. In opposition to the dramatic effect observed in PNT2‐ERG cells, in VCaP cells, the combination therapy did not show to significantly impair 3D cell growth, suggesting that the Erlotinib/TTI‐101 efficacy observed in early ERG‐driven PCa cell models may be lost with disease progression, with cells becoming “EGFR/STAT3 independent.” In line with this, stimuli with EGF in VCaP cells did not change p‐EGFR expression, supporting the hypothesis that other HER receptors, namely, HER2 and HER3, which heterodimerize with EGFR [46], may be activated upon EGF stimuli in ERG‐overexpressing PCa cells. Nevertheless, considering that higher synergism scores were obtained for VCaP cells with higher concentrations of EGFR and/or TTI‐101, the potential utility of this drug combination for the treatment of ERG‐positive PCa deserves further investigation.

Additionally, considering a recent study reporting activating mutations in ETV1 as possible drivers of NSCLC progression and resistance to anti‐EGFR therapy [47], it is also likely that the relevance of the identified ETV1‐EGFR/STAT3 signaling axis may extend beyond prostate carcinomas, namely, to other carcinomas frequently exhibiting overexpression of ETV1 (e.g., gastrointestinal stromal tumors and melanomas) [48].

Collectively, our results support the existence of an ETV1‐EGFR‐STAT3 signaling axis in PCa cells with ETV1 overexpression, and the utility of repurposing Erlotinib in combination with a STAT3 inhibitor, may target with higher efficiency PCa cells with ETV1 overexpression. Although *in vitro* cell models do not fully capture the complexity of PCa, the *in vitro* assessment of the impact of a coinhibition therapy targeting EGFR and STAT3 is aligned with the mechanistic observations in the same cell models, providing a rationale for its therapeutic efficacy in ETV1‐positive PCa. As Erlotinib is already FDA‐approved for the treatment of certain types of HNSCC, NSCLC and pancreatic cancer [49], its repurposing can significantly expedite clinical trials testing combined inhibition with TTI‐101 in PCa. In fact, clinical trials involving Erlotinib have shown some potential for clinical benefit in PCa, particularly in specific patient populations, but overall efficacy remains limited [50, 51]. The STAT3 inhibitor TTI‐101, while not yet FDA‐approved, has shown promise in preclinical studies and clinical trials for various cancers [52, 53]. Leveraging Erlotinib's established safety profile and dosing regimens, along with accumulating data on TTI‐101's pharmacokinetics and pharmacodynamics, could streamline the design of treatment protocols to enhance PCa patient management strategies. Future studies should explore *in vivo* models to confirm the ETS‐dependencies and define therapeutic schemes for clinical interventions.

## 5 Conclusions

Our study unveils EGFR‐STAT3 as an axis of oncogenic signaling driven by overexpression of the ETS transcription factor ETV1 and provides supporting evidence for the therapeutic potential of combining EGFR and STAT3 inhibitors in targeting (prostate) carcinomas with ETV1 overexpression. These findings highlight the promise of a tailored therapeutic approach for ETV1‐positive PCa and advocate for further exploration of this combination therapy in clinical trials. The potential utility of this combination scheme for ERG‐positive PCa requires further investigation.

## Conflict of interest

The authors declare no conflict of interest.

## Author contributions

Study conceptualization and design: PP; data collection: AA, AB, BO, EGP; analysis and interpretation of results: BO, EGP, and PP; manuscript writing: BO, EGP, PP, and MRT. All authors have read and agreed to the published version of the manuscript.

## Peer review

The peer review history for this article is available at https://www.webofscience.com/api/gateway/wos/peer‐review/10.1002/1878‐0261.70069.

## Supporting information


**Fig. S1.** ETS subtyping of TCGA‐PRAD samples.
**Fig. S2.** Automated high‐throughput quantification of cell viability and apoptosis in 2D models of prostate cell lines.
**Fig. S3.** Generating 3D spheroid models of tumorigenic LNCaP and VCaP cells.
**Fig. S4.** Validation of the association between EGFR/STAT3 and ETV1 expression in publicly available data from the GEO DataSets.
**Fig. S5.** Effect of EGF stimulation on ETV1 or ERG expression in PNT2‐derived and VCaP cell models.
**Fig. S6.** IC50 values of TTI‐101 and Erlotinib in the tumorigenic 22Rv1 cells.
**Fig. S7.** Synergism analysis using *Combenefit* software.
**Fig. S8.** Combined treatment effects on cell growth and apoptosis in ETV1 and ERG‐overexpressing cells.
**Table S1.** Preclinical and clinical data supporting the efficacy of the EGFR or STAT3 inhibitors used in this study in prostate cancer or other carcinomas.

## Data Availability

The data presented in this study are available upon request from the corresponding author.
