# Supplementary material for: EGFR‐STAT3 activation provides a therapeutic rationale for targeting aggressive ETV1‐positive prostate cancer
Source: Mol Oncol. 2025 Aug 14;19(11):3287–304. doi: 10.1002/1878-0261.70069 (PMC12591323; doi:10.1002/1878-0261.70069)
Supplement: Supplementary file 1 — Fig. S1. ETS subtyping of TCGA‐PRAD samples. Fig. S2. Automated high‐throughput quantification of cell viability and apoptosis in 2D models of prostate cell lines. Fig. S3. Generating 3D spheroid models of tumorigenic LNCaP and VCaP cells. Fig. S4. Validation of the association between EGFR/STAT3 and ETV1 expression in publicly available data from the GEO DataSets. Fig. S5. Effect of EGF stimulation on ETV1 or ERG expression in PNT2‐derived and VCaP cell models. Fig. S6. IC50 values of TTI‐101 and Erlotinib in the tumorigenic 22Rv1 cells. Fig. S7. Synergism analysis using Combenefit software. Fig. S8. Combined treatment effects on cell growth and apoptosis in ETV1 and ERG‐overexpressing cells. Table S1. Preclinical and clinical data supporting the efficacy of the EGFR or STAT3 inhibitors used in this study in prostate cancer or other carcinomas. [file MOL2-19-3287-s001.docx]

**Supporting Information**

Elsa Gomes Paiva^1,2,†^, Bernardo Orr^1,†^, Ana Azeredo^1,3,†^, Andreia Brandão^1^, Manuel R. Teixeira^1,4,5^ and Paula Paulo^1,5,*^

^1^Cancer Genetics Group, IPO Porto Research Center (CI-IPOP)/RISE@CI-IPOP (Health Research Network), Portuguese Oncology Institute of Porto (IPO Porto)/Porto Comprehensive Cancer Center, Raquel Seruca (Porto.CCC Raquel Seruca), 4200-072 Porto, Portugal

^2^PhD Program in Biomedical Sciences, School of Medicine and Biomedical Sciences (ICBAS), University of Porto, 4050-313 Porto, Portugal

^3^Master Program in Oncology, School of Medicine and Biomedical Sciences (ICBAS), University of Porto, 4050-313 Porto, Portugal

^4^Department of Laboratory Genetics, Portuguese Oncology Institute of Porto (IPO Porto)/Porto Comprehensive Cancer Center Raquel Seruca (Porto.CCC Raquel Seruca), 4200-072 Porto, Portugal

^5^School of Medicine and Biomedical Sciences (ICBAS), University of Porto, 4050-313 Porto, Portugal

^†^Authors contributed equally to this work

^*^Correspondence to paula.paulo@ipoporto.min-saude.pt (+351225084000, ext. 5609)

**Running title:** Targeting EGFR-STAT3 in ETV1-positive prostate cancer

**Keywords:** ETV1 rearrangements/overexpression, feedback loop, EGFR/STAT3 activation, Erlotinib, TTI-101, prostate cancer

**Supporting Tables**

**Supporting Table 1 –** Preclinical and clinical data supporting the efficacy of the EGFR or STAT3 inhibitors used in this study in prostate cancer or other carcinomas.

| Drug | Target | Preclinical studies | Clinical trials |
| --- | --- | --- | --- |
| Erlotinib | EGFR | (1,2) | (3,4) |
| TTI-101 | STAT3 | (5,6,7) | (8) |

**Supporting Figures**

**
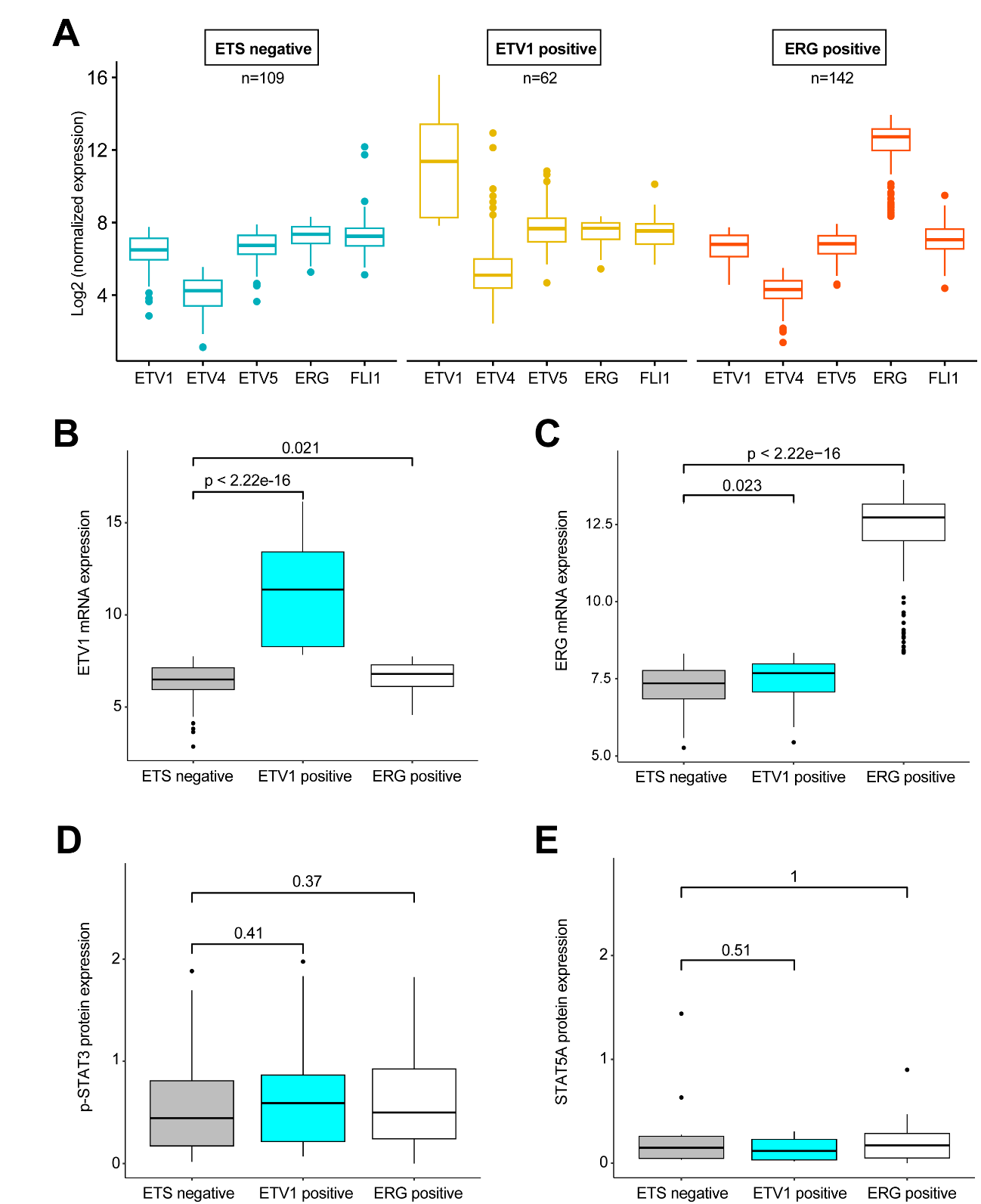
**

**Supporting Figure 1 – ETS subtyping of TCGA-PRAD samples.** **(A)** Box-plot distribution of the expression levels of ETV1, ETV4, ETV5, ERG and FLI1 for the three ETS molecular subgroups – “ETS negative”, “ETV1 positive” and “ERG positive” – defined for the TCGA-PRAD data collection; y-axis indicates normalized relative mRNA expression. **(B-E)** Analysis of ETV1 mRNA expression **(B)**, ERG mRNA expression **(C)**, p-STAT3 **(D)**, and STAT5A protein expression levels **(E)** in the defined “ETS negative”, “ETV1 positive” and “ERG positive” tumors; y-axis indicates normalized relative expression.

**
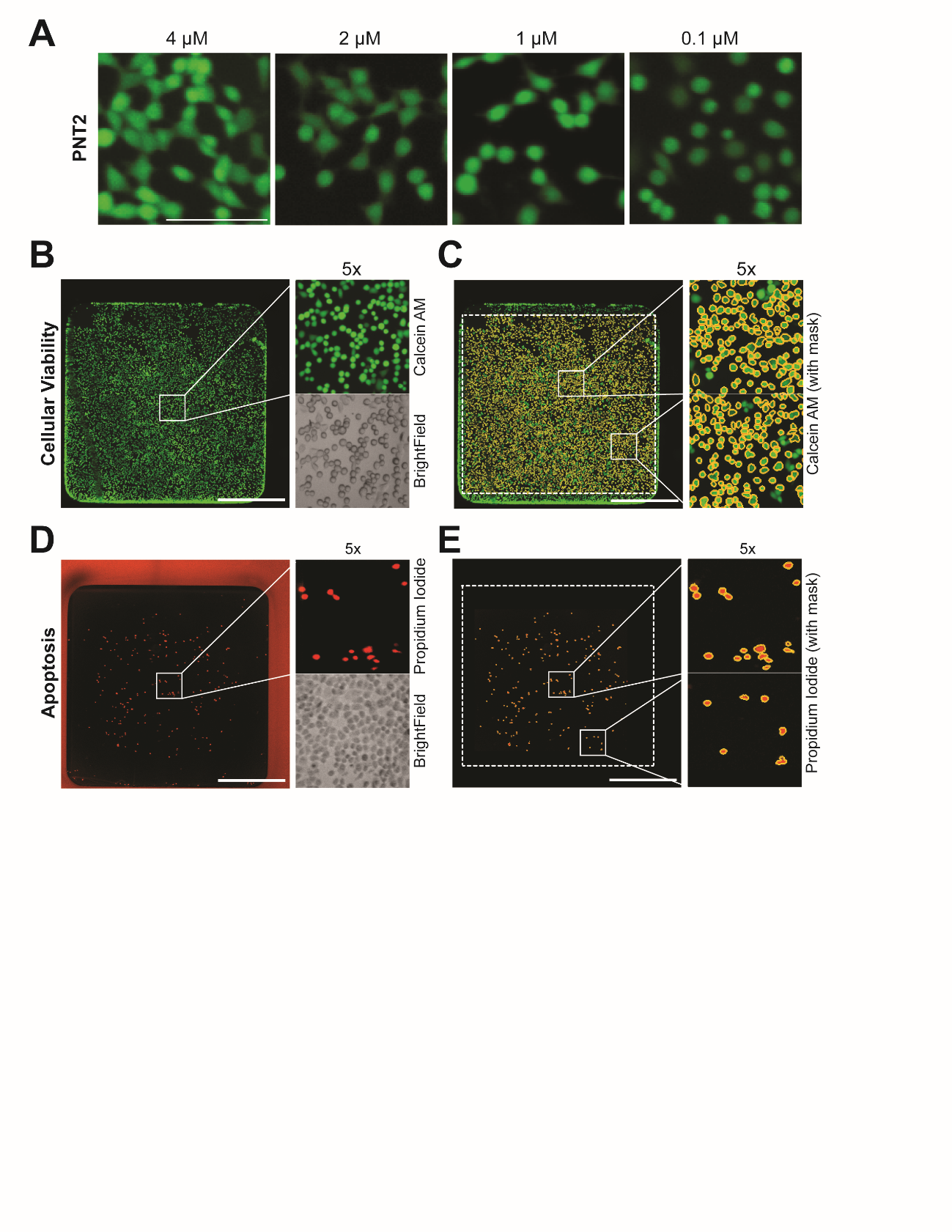
**

**Supporting Figure 2 – Automated high-throughput quantification of cell viability and apoptosis in 2D models of prostate cell lines.** **(A)** Representative images of PNT2 cells treated with varying concentrations of Calcein AM, demonstrating restricted Calcein AM localization to nuclei using 0.1µM Calcein AM. Scale bar = 100µm. **(B, D)** Representative images of PNT2 cells growing in a 384-well plate showing viable cells labeled with Calcein AM **(B)** and apoptotic cells labeled with Propidium Iodide (PI) **(D)**. Insets represent 5X magnifications of selected regions showing BrightField and Calcein AM/PI signals. **(C, E)** Representative image showing masks (yellow) generated using Gen5 software that were used for automated quantification of viable (Calcein AM stained) **(C)** and apoptotic (PI stained) **(E)** cells. Dashed white lines delineate the area of analysis and insets represent 5X magnifications of selected regions. Scale bar = 1000µm.

**
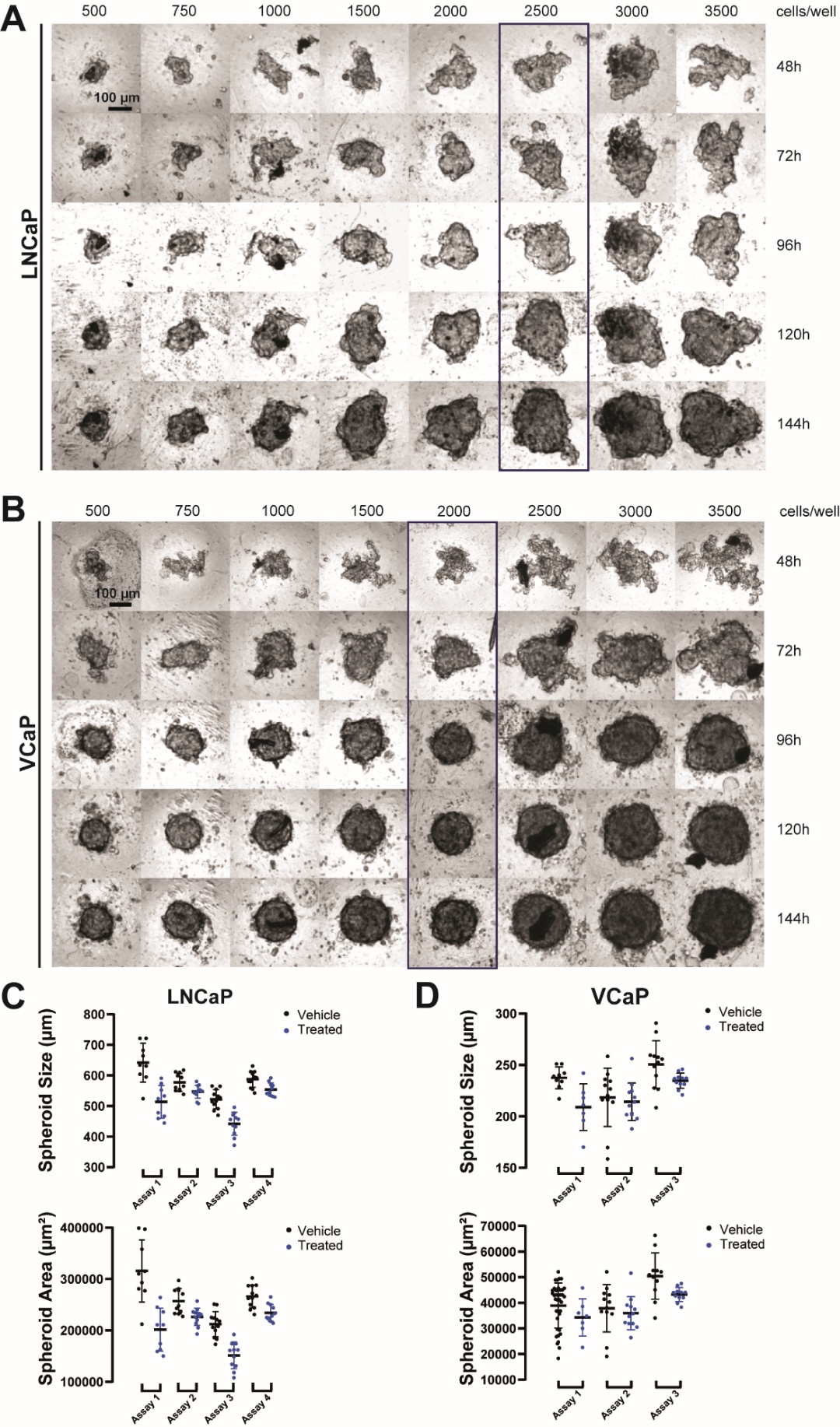
**

**Supporting Figure 3 – Generating 3D spheroid models of tumorigenic LNCaP and VCaP cells.** **(A, B)** Panel showing representative spheroids’ images of LNCaP **(A)** and VCaP **(B)** cells, seeded at different cell densities in ultra-low attachment 384-well plates for 6 days. Scale bar = 100 µm. **(C, D)** Quantification of spheroid size and area for LNCaP **(C)** and VCaP **(D)** cells after 96 hours of combined treatment, represented as mean spheroid diameter (µm) ± standard deviation and mean spheroid area (µm²) ± standard deviation, respectively.

**
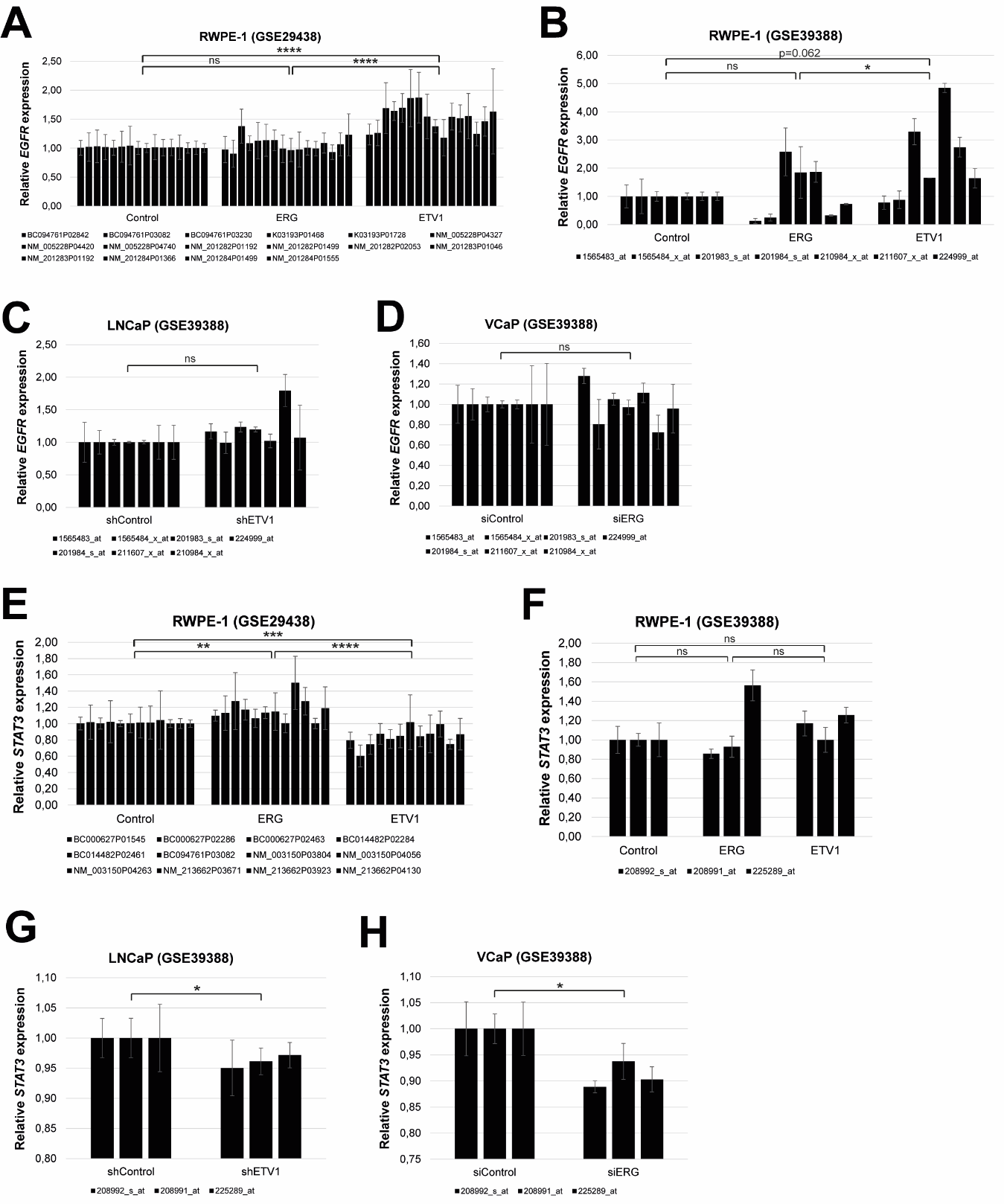
**

**Supplorting Figure 4 – Validation of the association between EGFR/STAT3 and ETV1 expression in publicly available data from the GEO DataSets. (A-D)** EGFR expression data obtained from the GSE29438 **(A)** and GSE39388 **(B)** DataSets for RWPE1 control and RWPE1-derived cells overexpressing ETV1 or ERG**,** and for LNCaP **(C)** and VCaP **(D)** cells with silencing of ETV1 or ERG, respectively. **(E-H)** STAT3 expression data obtained from the GSE29438 **(E)** and GSE39388 **(F)** DataSets for RWPE1 control and RWPE1-derived cells overexpressing ETV1 or ERG**,** and for LNCaP **(G)** and VCaP **(H)** cells with silencing of ETV1 or ERG, respectively. Each bar represents data for a different microarray probeset covering the different EGFR/STAT3 transcripts, and error bars represent the standard deviation from triplicate or quadruplicate biologic replicates. The probes are ordered accordingly from left to right. To assess statistical differences, the paired t-test was used.

**
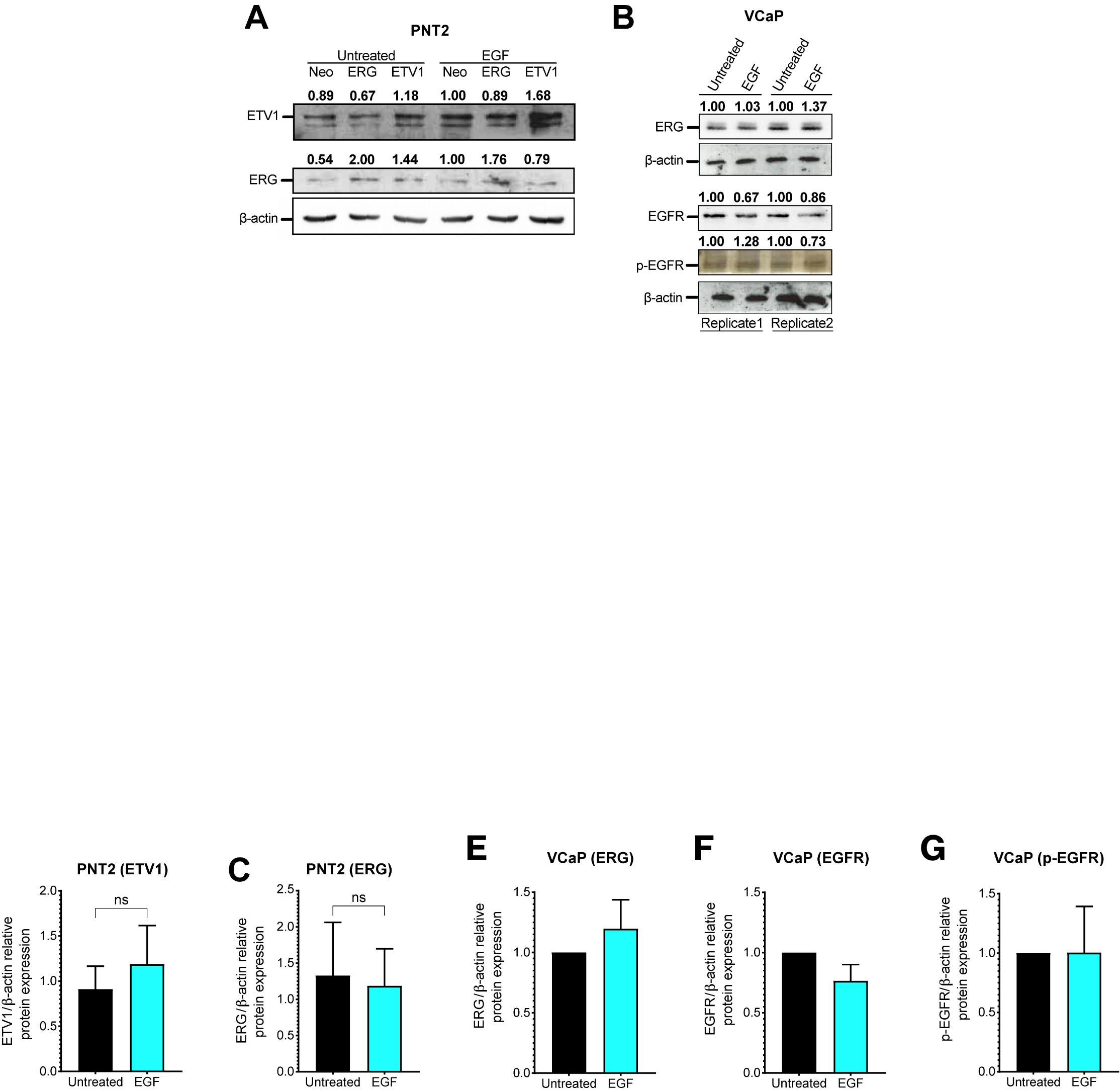
**

**Supporting Figure 5 – Effect of EGF stimulation on ETV1 or ERG expression in PNT2-derived and VCaP cell models. (A)** Representative protein blots showing ETV1 and ERG expression in PNT2 control (Neo) and PNT2-derived cells overexpressing ETV1 (ETV1) or ERG (ERG), under untreated conditions or after EGF stimulation. β-actin was used as a loading control. ETV1 expression is increased after EGF stimuli in all cell models, while ERG expression is not. **(B)** Representative protein blots showing ERG, EGFR, and phosphorylated EGFR (p-EGFR) expression in VCaP cells, under untreated conditions or after EGF stimulation. β-actin was used as a loading control. Two independent replicates shown. EGF stimuli does not consistently impacts ERG or p-EGFR expression, while EGFR expression seems to be decreased after EGF stimuli.


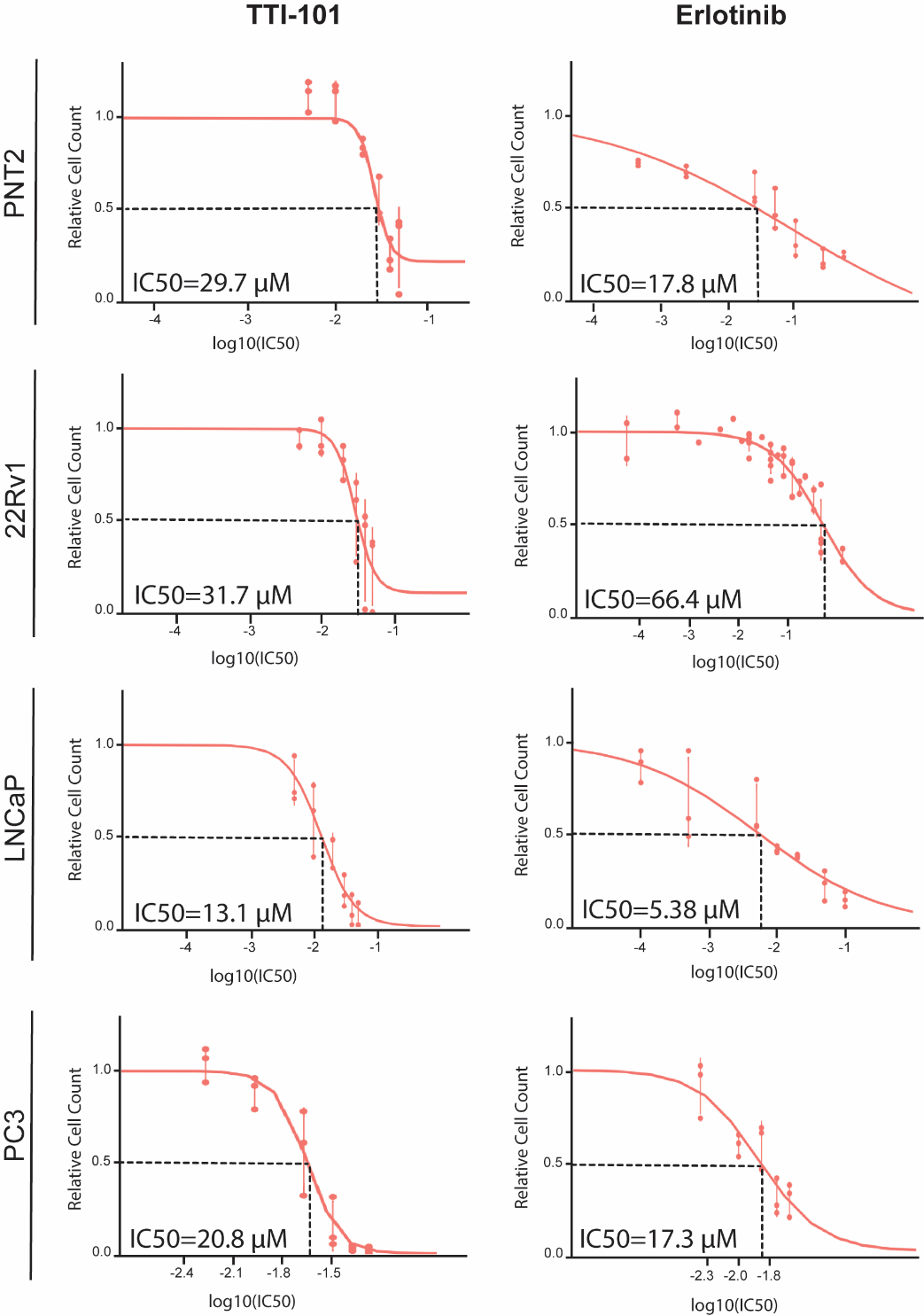


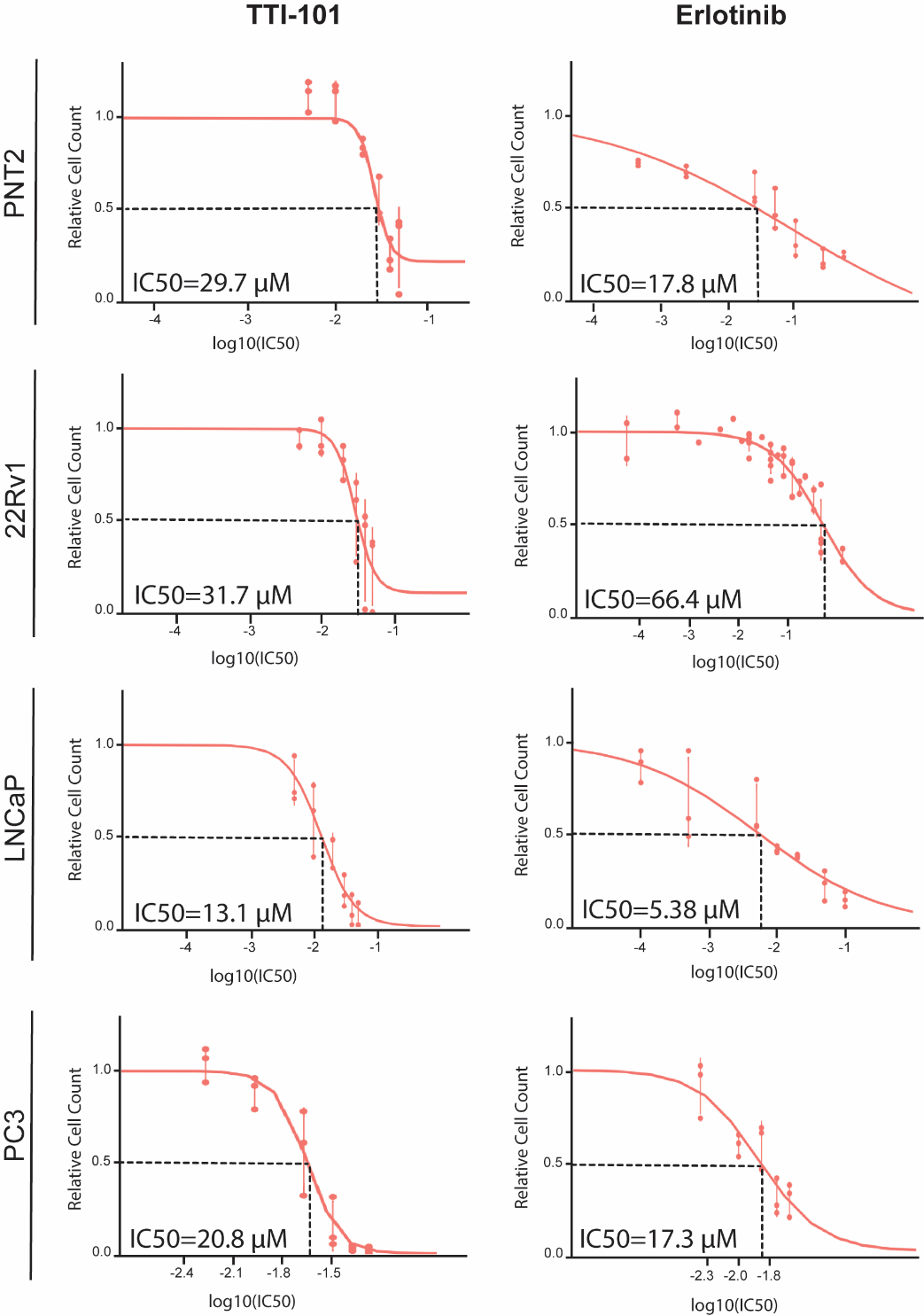


**Supporting Figure 6 – IC50 values of TTI-101 and Erlotinib in the tumorigenic 22Rv1 cells.** IC50 concentrations were determined with data obtained from MTT-based cell viability assays after 48-hour treatments using the GR online calculator (9). Error bars represent the standard deviation from three independent experiments.

**
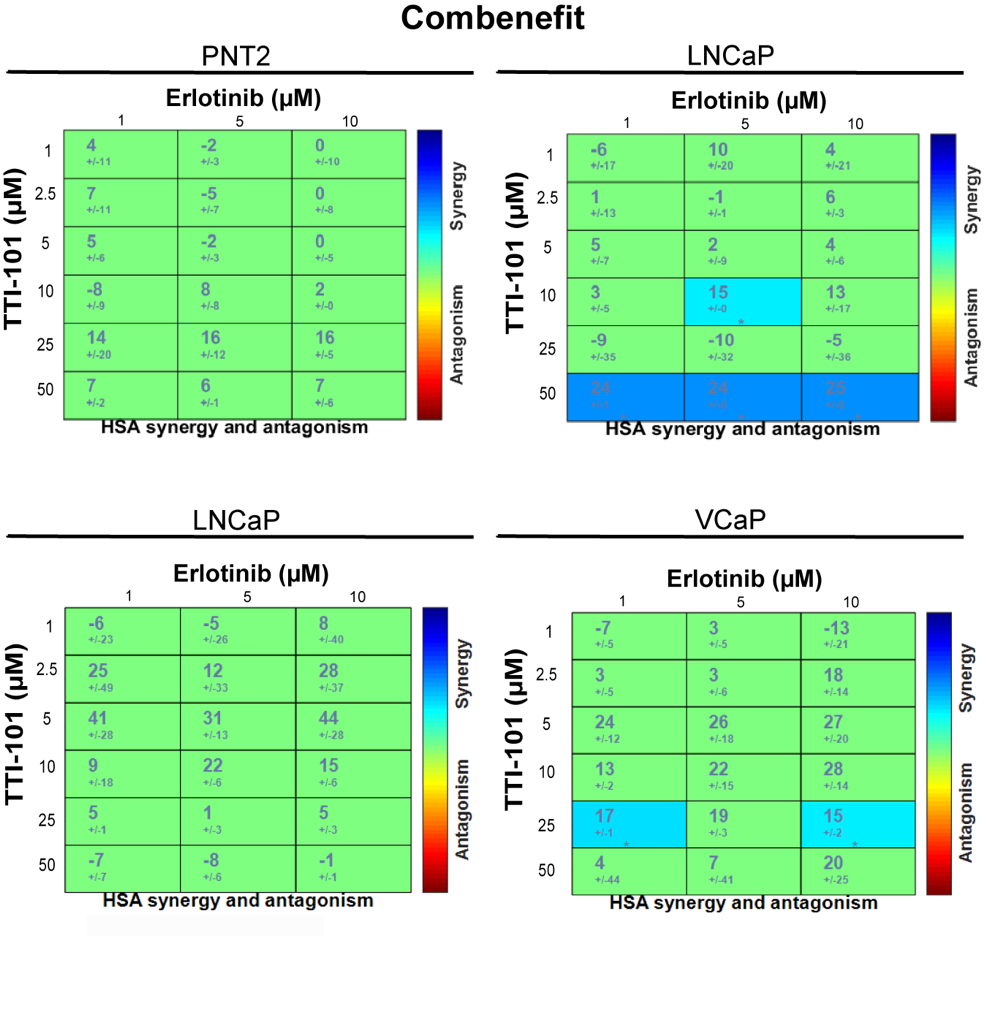
A**

**B**

**Supporting Figure 7 – Synergism analysis using *Combenefit* software. A)** Heat-maps representing the impact on cell viability imposed by treatments with different dose combinations of TTI-101 and Erlotinib in PNT2 and LNCaP cell models, in 96-well plates. **B)** Heat-maps representing the impact on cell viability imposed by treatments with different dose combinations of TTI-101 and Erlotinib in LNCaP and VCaP cell models, in 384-well plates. The input data was the same as used with *SynergyFinder+*. For each drug combination a synergy score is shown: negative values (pointing to antagonistic effect) are shown in yellow-to-red colors, and positive values (pointing to synergistic effect) are shown in green-to-blue colors (10). Data represent mean ± SD from two independent experiments.

**
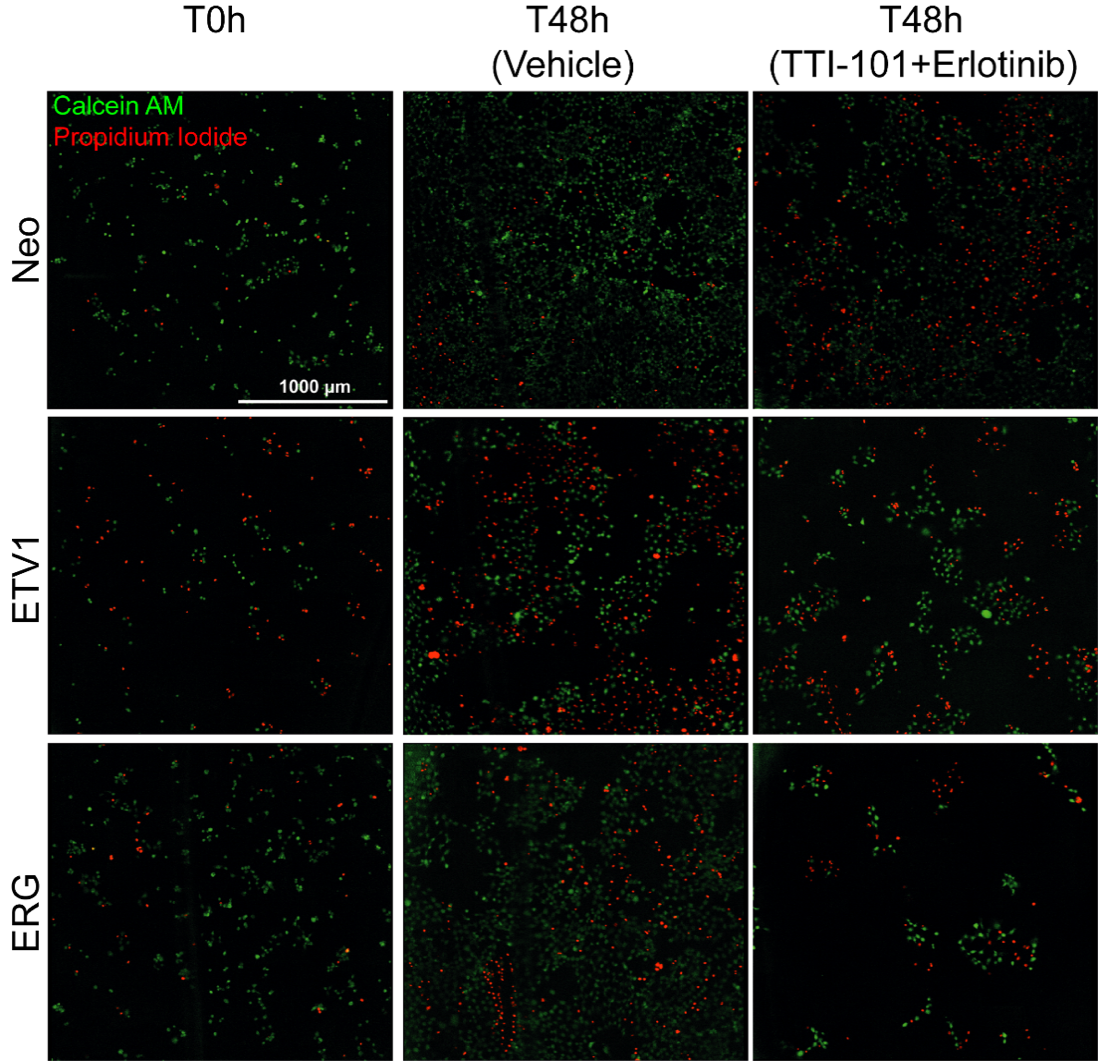
**

**Supporting Figure 8 – Combined treatment effects on cell growth and apoptosis in ETV1 and ERG-overexpressing cells.** **(A)** Representative images of PNT2 Neo cells at baseline (0 hours, T0h), and after 48 hours of treatment with either vehicle (0.1% DMSO) or a combination of TTI-101+Erlotinib (10 µM and 5 µM, respectively). Cells were stained with Calcein AM (green) to label live cells and Propidium Iodide (red) to label dead cells. Images were acquired using *Gen5* software for automated quantification. **(B, C)** Representative images of ETV1-overexpressing **(B)** and ERG-overexpressing **(C)** cells under the same treatment conditions. Scale bar = 1000 µm.

**References**

1. Qi W, Cooke LS, Stejskal A, Riley C, Croce KD, Saldanha JW, Bearss D, Mahadevan D. MP470, a novel receptor tyrosine kinase inhibitor, in combination with Erlotinib inhibits the HER family/PI3K/Akt pathway and tumor growth in prostate cancer. BMC Cancer. 2009;9:142.

2. Festuccia C, Gravina GL, Biordi L, D'Ascenzo S, Dolo V, Ficorella C, Ricevuto E, Tombolini V. Effects of EGFR tyrosine kinase inhibitor erlotinib in prostate cancer cells in vitro. Prostate. 2009;69(14):1529-37.

3. Gravis G, Bladou F, Salem N, Gonçalves A, Esterni B, Walz J, Bagattini S, Marcy M, Brunelle S, Viens P. Results from a monocentric phase II trial of erlotinib in patients with metastatic prostate cancer. Ann Oncol. 2008;19(9):1624-8.

4. Nabhan C, Lestingi TM, Galvez A, Tolzien K, Kelby SK, Tsarwhas D, et al. Erlotinib has moderate single-agent activity in chemotherapy-naïve castration-resistant prostate cancer: final results of a phase II trial. Urology. 2009;74(3):665-71.

5. Kong R, Sun G, Li X, Wu L, Li L, Li Y, et al. Small Molecule Inhibitor C188-9 Synergistically Enhances the Demethylated Activity of Low-Dose 5-Aza-2′-Deoxycytidine Against Pancreatic Cancer. Frontiers in Oncology. 2020;10:612.

6. Li Y, Dong Y. TTI-101 targets STAT3/c-Myc signaling pathway to suppress cervical cancer progression: an integrated experimental and computational analysis. Cancer Cell Int. 2024;24(1):286.

7. Singh SP, Pathuri G, Asch AS, Rao CV, Madka V. Stat3 Inhibitors TTI-101 and SH5-07 Suppress bladder cancer cell survival in 3D tumor models. Cells. 2024 Aug 31;13(17):1463. Erratum in: Cells. 2025;14(4):299.

8. Tsimberidou AM, Vining DJ, Arora SP, de Achaval S, Larson J, Kauh J, Cartwright C, Avritscher R, Alibhai I, Tweardy DJ, Kaseb AO. Phase I Trial of TTI-101, a First-in-Class Oral Inhibitor of STAT3, in Patients with Advanced Solid Tumors. Clin Cancer Res. 2025;31(6):965-74.

9. Clark NA, Hafner M, Kouril M, Williams EH, Muhlich JL, Pilarczyk M, et al. GRcalculator: an online tool for calculating and mining dose–response data. BMC Cancer. 2017;17(1):698.

10. Di Veroli GY, Fornari C, Wang D, Mollard S, Bramhall JL, Richards FM, Jodrell DI. Combenefit: an interactive platform for the analysis and visualization of drug combinations. 2016;32(18):2866-8.
